# Exploring the impact of different types of exercise on working memory in children with ADHD: a network meta-analysis

**DOI:** 10.3389/fpsyg.2025.1522944

**Published:** 2025-01-27

**Authors:** Xiangqin Song, Yaoqi Hou, Wenying Shi, Yan Wang, Feifan Fan, Liu Hong

**Affiliations:** College of Physical Education and Sports, Beijing Normal University, Beijing, China

**Keywords:** physical activity, working memory, children, attention deficit hyperactivity disorder, network meta-analysis

## Abstract

**Background:**

Attention-deficit hyperactivity disorder (ADHD) is a common neurodevelopmental disorder in children, often accompanied by working memory deficits. Recently, exercise interventions have gained attention as a potential strategy to improve cognitive function in children with ADHD. However, the effects of different types of exercise on working memory remain unclear. This study aimed to assess the effects of various exercise interventions on working memory in children with ADHD using a network meta-analysis.

**Methods:**

A comprehensive search was conducted in PubMed, Cochrane, Embase, and Web of Science databases for relevant studies. After screening according to the inclusion and exclusion criteria, a total of 17 eligible studies were identified for analysis. A network meta-analysis was performed to integrate data and evaluate the effects of cognitive-aerobic exercise, ball games, mind-body exercises, interactive games, and general aerobic exercise on working memory in children with ADHD.

**Results:**

The results indicated significant differences in the effectiveness of various types of exercise interventions on working memory in children with ADHD. Cognitive-aerobic exercise showed the most significant effect (SMD = 0.72, 95% CI: 0.44–1.00), followed by ball games (SMD = 0.61, 95% CI: −0.12–1.35). Mind-body exercises and interactive games had moderate effects (SMD = 0.50 and 0.37, respectively), while general aerobic exercise showed relatively small effects (SMD = 0.40, 95% CI: 0.19–0.60). SUCRA analysis further confirmed the highest preference for cognitive-aerobic exercise in improving working memory. Meta-regression analysis showed that intervention frequency and total intervention duration significantly affected the effectiveness of cognitive-aerobic exercise, while other variables did not significantly moderate the effects.

**Conclusion:**

Cognitive-aerobic exercise had the most significant effect on improving working memory in children with ADHD. Higher intervention frequency and longer intervention duration may enhance its effects. Future research should explore the impact of these factors and consider increasing sample sizes to validate the role of these moderators.

**Systematic review registration:**

https://www.crd.york.ac.uk/PROSPERO/display_record.php?RecordID=627915.

## 1 Introduction

Attention Deficit Hyperactivity Disorder (ADHD) is a neurodevelopmental disorder that has seen a rising prevalence in recent years. The prevalence in children and adolescents is approximately 5%–7%, with the diagnosis rate significantly higher in boys than in girls (Mohammadi et al., 2021). According to data from the Centers for Disease Control and Prevention (CDC), by 2020, approximately 9.4% of children aged 3 to 17 years were diagnosed with ADHD, which equates to nearly one in every 10 children being affected (Harris, 2024). Individuals with ADHD commonly exhibit difficulty maintaining attention, are easily distracted by external stimuli, show a lack of attention to detail, and struggle to organize and complete tasks, particularly those requiring sustained focus (Bush, 2010). Additionally, hyperactivity is often observed, such as an inability to sit still, frequent movement, climbing, or running, and engaging in hyperactive behaviors in inappropriate situations (de la Peña et al., 2020). ADHD patients tend to be impulsive, characterized by a lack of self-control, poor planning, frequent interruptions of others, and expressing opinions at inappropriate times (Ayano et al., 2023). In adults, symptoms may manifest as inner restlessness, difficulty remaining calm, and emotional dysregulation (Soler-Gutiérrez et al., 2023).

Working memory refers to the ability to hold and manipulate information over a short period and is a crucial component of executive function, supporting the completion of daily tasks, learning, and social interactions (Baddeley, 2012). In individuals with ADHD, impaired working memory leads to frequent distractions and difficulty maintaining attention during tasks (Kasper et al., 2012). This impairment manifests in several ways: interruptions during multi-step tasks, forgetfulness of important information, and challenges in emotional regulation and impulse control (Kofler et al., 2018; Ramos et al., 2020). Two primary theoretical models explain the relationship between ADHD and working memory. Barkley's response inhibition model suggests that deficient response inhibition is the primary impairment, leading to executive function deficits including working memory problems, which result in hyperactivity, impulsivity, and inattention symptoms (Barkley, 1997). The alternative working memory model, drawing from Baddeley and Hitch's multi-component theory, positions working memory deficits as a core feature of ADHD (Rapport et al., 2001). This model, based on Baddeley and Hitch's multi-component working memory theory, includes four elements: the central executive system, phonological loop, visuospatial sketchpad, and the episodic buffer, which work together to coordinate the storage and processing of information (RepovŠ and Baddeley, 2006; Baddeley, 1996, 1992). Deficits in working memory pose significant challenges for ADHD patients in academic, social, and emotional management. Improving working memory in ADHD patients is considered key to enhancing their overall cognitive function (Rogers et al., 2011). Given these significant impacts of working memory deficits on ADHD symptoms, researchers have investigated various interventions to address these challenges, with physical exercise emerging as a particularly promising approach.

Exercise refers to a subset of physical activity that is planned, structured, repetitive, and purposive, with the objective of improving or maintaining physical fitness and overall health. Unlike general physical activity, such as walking or casual playing, exercise involves deliberate effort to achieve specific fitness goals (Bouchard et al., 2012). This definition encompasses a wide range of activities, including aerobic exercises, strength training, flexibility exercises, and motor skill-based training. In the context of this study, we focus on aerobic exercises, which are defined as sustained physical activities that increase heart rate and improve cardiovascular fitness (Kargarfard and Afrasyabi, 2015; Mehren et al., 2019). These exercises can further be classified into two main categories: traditional aerobic exercise and cognitively enriched aerobic exercise. Traditional aerobic exercise involves repetitive and rhythmic movements, such as running, swimming, or cycling, aimed solely at improving physical endurance and fitness. Cognitively enriched aerobic exercise, on the other hand, combines physical activity with simultaneous cognitive tasks, such as decision-making, problem-solving, or memory challenges (Karssemeijer et al., 2017; Shimada et al., 2018). Examples include dual-task exercises, strategy-based games, and exergaming (Lauenroth et al., 2016). By distinguishing between these two types of aerobic exercise, this study aims to investigate their differential impacts on working memory in children with ADHD, emphasizing the potential added benefits of cognitive engagement during physical activity.

Exercise interventions have been shown in numerous studies to have a positive impact on working memory (Chaire et al., 2020; Zhidong et al., 2021). Physical exercise, especially aerobic exercise and moderate-intensity physical activity, is believed to promote brain plasticity, enhance neurotransmission related to memory and learning (such as the levels of dopamine and norepinephrine), and improve cerebral blood flow and oxygen supply, thereby facilitating the enhancement of working memory (Pickersgill et al., 2022; Johansson et al., 2022). Studies have found that regular physical activity, particularly exercise forms that involve attentional demands, such as cognitive-motor skill exercises, not only improve working memory capacity but also enhance information processing speed and cognitive flexibility (Hou et al., 2024). Research indicates that aerobic exercises such as running, swimming, and cycling can significantly alleviate symptoms of hyperactivity, impulsivity, and inattention in children with ADHD (Feng et al., 2024; Sun et al., 2022). Exercise not only helps release neurotransmitters like dopamine and norepinephrine in the brain but also regulates mood and improves behavioral control (Bastioli et al., 2022). These effects significantly contribute to enhancing academic performance and social skills in children with ADHD, while the feasibility and enjoyment of physical activities make them more easily integrated into their daily routines.

Although existing studies have confirmed the positive impact of exercise interventions on working memory in children with ADHD, differences in intervention effects may exist across various types of exercise, such as aerobic exercise, cognitive exercise, and skill-based exercises. However, most current studies have focused on the effects of individual exercise forms, and comparative research on multiple types of exercise interventions is relatively scarce. This limitation makes it difficult for researchers to comprehensively understand which exercise modality has the most beneficial effect on improving working memory in children with ADHD. Therefore, it is necessary to adopt a comprehensive approach that integrates the effects of different exercise interventions, providing a more comprehensive reference for intervention strategies.

Therefore, it is necessary to adopt a comprehensive approach that integrates the effects of different exercise interventions, providing a more comprehensive reference for intervention strategies. Based on previous literature and theoretical frameworks, we proposed the following hypotheses: Different types of exercise interventions would show varying degrees of effectiveness in improving working memory, with structured exercise programs showing greater benefits than general physical activity. Exercise interventions that involve higher levels of cognitive engagement and motor skill development would lead to greater improvements in working memory performance compared to simple aerobic exercises, due to their enhanced effects on neural plasticity and neurotransmitter regulation.

## 2 Method

### 2.1 Protocol

This study was conducted according to the Preferred Reporting Items for Systematic Reviews and Network Meta-Analyses (PRISMA-NMA) guidelines. The PRISMA checklist is provided in Appendix. PROSPERO registration number CRD42024627915.

### 2.2 Search strategy

The search strategy was developed based on the PICOS framework and conducted through systematic searches in the PubMed, Embase, Cochrane Library, and Web of Science (WoS) databases to ensure coverage of major biomedical, psychological, and multidisciplinary research sources. The inclusion time frame for the literature will cover from the establishment of each database to November 2024. Key terms included ADHD, exercise intervention, children and adolescents, and working memory, combined using Boolean logic terms (AND, OR) to obtain literature highly relevant to the research topic. Additionally, during the search process, manual screening of individual searches or the extraction of relevant studies from published meta-analyses was performed to supplement and refine the data sources. Detailed search strategies, subject terms, and keywords can be found in the Appendix. See Appendix for further details.

### 2.3 Inclusion and exclusion criteria

#### 2.3.1 Inclusion criteria

The inclusion criteria for this study were as follows: participants were children and adolescents aged 3 to 18 years diagnosed with ADHD based on internationally recognized diagnostic criteria, such as DSM-5 or ICD-10, ensuring consistency and accuracy in ADHD diagnosis. The interventions included structured exercise programs, such as aerobic exercise (e.g., running, swimming), strength training (e.g., resistance training), cognitive exercise, and balance or coordination exercises, with clearly reported duration, frequency, and intensity to ensure standardization and reproducibility. The studies must include a control group, such as no-intervention groups, routine care, or non-exercise intervention groups (e.g., behavioral therapy), or comparisons between different exercise interventions with sufficient distinction. The primary outcome was required to focus on changes in working memory, measured using standardized and validated tools, with a clear distinction from other cognitive function indicators when multiple outcomes were reported. Eligible study designs included randomized controlled trials (RCTs). Studies were excluded if they involved participants with severe neurological, psychiatric, or physical disorders unrelated to ADHD, focused solely on pharmacological or psychological therapies without an exercise component, lacked adequate control groups, did not report working memory-specific outcomes, or were published in languages other than English or provided insufficient data for analysis.

#### 2.3.2 Exclusion criteria

Research subjects meeting any of the following criteria will be excluded: (1) presence of severe mental or neurological disorders that may affect cognitive function; (2) ADHD diagnosis based solely on teacher or parent reports without clinical evaluation; (3) interventions where the independent effects of exercise cannot be isolated from other treatment components; (4) studies where control groups received mixed interventions for ethical or other considerations; (5) interventions lacking clear documentation of exercise instructor qualifications or professional credentials; (6) studies without reported participant compliance or completion rates; (7) research with significant methodological bias or inappropriate application of measurement instruments; and (8) case studies, retrospective analyses, or review articles. Additionally, studies showing significant baseline differences between experimental and control groups without proper statistical adjustment will be excluded.

### 2.4 Literature screening and data extraction

During the literature screening and data extraction phase, the retrieved studies will first be imported into the EndNote reference management software for duplicate removal. Subsequently, two rounds of literature screening will be conducted according to the pre-established inclusion and exclusion criteria. In the first round, screening will be based on titles and abstracts, and will be performed by two independent reviewers to exclude studies that clearly do not meet the criteria. The second round will involve a full-text review of the studies that passed the initial screening to ultimately determine which studies meet the inclusion criteria. In cases of disagreement between the two reviewers during the screening process, the issue will be resolved through discussion or arbitration by a third reviewer.

For data extraction, a pre-designed data extraction form will be used to collect relevant information, including but not limited to: basic study information (such as authors, publication year, country), study design type, sample characteristics (such as age, gender, and ADHD diagnostic criteria), detailed descriptions of the intervention (such as type of exercise, intensity, frequency, and duration), control group information, outcome measures, and main results (quantitative data on working memory). Data extraction will be conducted independently by two reviewers, with cross-checking to ensure accuracy and consistency. If any data is missing or unclear, attempts will be made to contact the authors of the original studies for clarification.

### 2.5 Quality assessment of the literature

To systematically assess the methodological quality of the included studies, this research will use the Physiotherapy Evidence Database (PEDro) scale to evaluate the quality of each randomized controlled trial (RCT). The PEDro scale consists of 11 criteria designed to identify potential biases in study design, covering aspects such as random allocation, baseline similarity, blinding, and outcome measurement. Studies with a score below 4 will be considered to have a high risk of methodological bias and will be excluded from the analysis; studies with a score above 4 will be classified according to score levels to ensure that only high-quality studies are included, thereby improving the reliability of the results. For non-randomized controlled trials (non-RCTs), this research will use the Methodological Index for Non-Randomized Studies (MINORS) scale to evaluate methodological quality. The MINORS scale addresses the specific requirements for non-randomized studies, including reporting baseline characteristics, describing sample selection, and assessing primary outcomes. Similar to the PEDro scale, the MINORS scale ensures a comprehensive evaluation of methodological rigor and potential biases. The use of these two scales ensures that the analysis is based on high-quality evidence, minimizing the impact of methodological biases on the study conclusions.

### 2.6 Risk of bias assessment

The risk of bias assessment was conducted using tools recommended by Cochrane. For randomized controlled trials (RCTs), the Risk of Bias 2 (RoB 2) tool was employed to evaluate five domains: randomization process, intervention implementation, missing data, outcome measurement, and outcome reporting. Studies were rated as “low risk” if all domains were assessed as low risk, “some concerns” if at least one domain indicated concerns but none were high risk, and “high risk” if at least one domain was high risk or four or more domains indicated concerns. For non-randomized studies, the Risk Of Bias In Non-randomized Studies – of Interventions, Version 2 (ROBINS-I V2) tool was utilized to assess seven domains: confounding, participant selection, intervention classification, intervention implementation, missing data, outcome measurement, and outcome reporting. The risk of bias was categorized as “low,” “moderate,” or “serious.” The integration of results from both tools enabled a comprehensive evaluation of the evidence quality in this study.

### 2.7 Data analysis

In this study, the network meta-analysis will be conducted using R software (version 4.3.2) with the “gemtc” package, combined with the JAGS program, through the Bayesian Markov Chain Monte Carlo (MCMC) algorithm under a random-effects model for data synthesis. To ensure the accuracy of the analysis, four Markov chains will be used for model construction, with 20,000 iterations, the first 5,000 of which will be used for preliminary annealing to promote model convergence. The effectiveness of model convergence will be assessed using the Potential Scale Reduction Factor (PSRF), with a PSRF value close to 1 indicating satisfactory convergence. In the calculation of effect sizes, standardized mean differences (SMDs) and their 95% confidence intervals (CIs) will be used to accommodate variations in measurement tools across the included studies. According to Cohen's classification, an SMD ≥ |0.8| is considered a large effect, ≥|0.5| but < |0.8| as a medium effect, ≥|0.2| but < |0.5| as a small effect, and < |0.2| is regarded as a negligible effect (Chen et al., 2010).

The initial steps of the analysis will involve constructing a network evidence plot to visualize the direct and indirect relationships between different exercise interventions. The thickness of the lines in the plot will be proportional to the number of related studies, and interventions with larger sample sizes will be represented by larger nodes. For closed-loop evidence networks, consistency testing will be performed using the node-splitting method. If the *P*-value exceeds 0.05, it indicates good consistency between the interventions. For interventions without direct comparisons, indirect assessments will be made using the network meta-analysis framework. Additionally, the cumulative ranking (SUCRA) values for each intervention comparison will be calculated to rank their effects. A SUCRA value close to 1 indicates a better effect, while a value closer to 0 indicates a poorer effect.

## 3 Results

**Figure 1:**
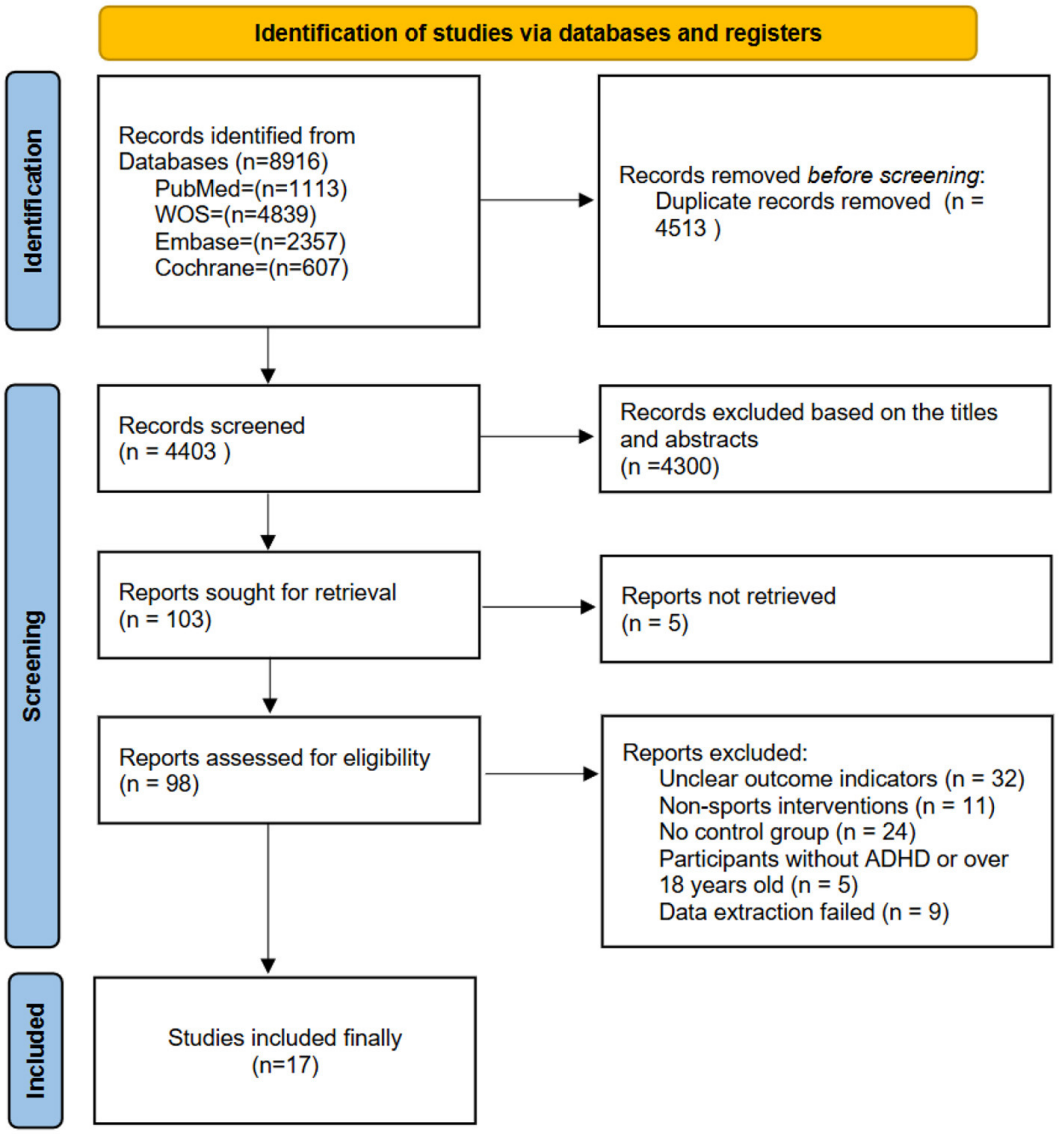
Flowchart of literature screening.

In the literature screening process of this study, a total of 8,916 relevant articles were retrieved from multiple databases (PubMed, WOS, Embase, Cochrane). After removing duplicates, 4,403 articles remained. During the title and abstract screening, 4,513 irrelevant articles were excluded, leaving 98 articles for full-text review. In the full-text screening, 32 articles were excluded due to unclear outcome measures, 11 articles were excluded for being non-exercise interventions, 24 articles were excluded for lacking control groups, 5 articles were excluded due to non-ADHD participants or age outside the inclusion range, and 9 articles were excluded because the data could not be extracted. Ultimately, 17 studies that met the inclusion criteria were included. The literature search flow is shown in Figure 1.

The 17 included studies involved 419 participants, with intervention periods ranging from 1 to 13 weeks. The frequency of intervention was between 1 and 5 times per week, with each session lasting from 10 to 90 min. Participants' ages ranged from 3 to 18 years, with a relatively low proportion of female participants, and some studies only included male participants. The included studies were conducted across several countries, including China (5 studies), the United States (3 studies), Switzerland (2 studies), Iran (2 studies), the Netherlands (3 studies), Germany (1 study), and Canada (1 study). Various exercise interventions were used in the experimental groups, such as cycling, judo training, motion-sensing games, yoga, and aerobic exercise combined with cognitive tasks. The control groups were set as no treatment, regular education, watching videos, sedentary activities, etc. Multiple outcome measurement tools were used to assess work memory and other cognitive metrics. Table 1 provides a detailed summary of the basic characteristics of each study, including the first author, publication year, country, age range, mean age, proportion of females, experimental design, intervention duration, and outcome measurement tools.

**Table 1:** Basic characteristics of the included studies.

**References**	**Country**	**Sample age (years)**	**Male percentage (%)**	**Sample size (E/C)**	**Experimental group**	**Control group**	**Intervention dose**	**Outcome indicator**	**Diagnostic**
Van Riper et al. (2023)	USA	Mean: 10–18 Range: 13.6 ± 1.9	73.30%	28 (15/23)	Cycling	No treatment	25 min ^*^ 1 times ^*^ 1 weeks	GNG N-Back	Hospital certificate
Ludyga et al. (2022)	China	Mean: 8–12 Range: 10.8 ± 1.2	NA	57 (29/28)	Judo Training	Regular education	60 min ^*^ 2 times ^*^ 12 weeks	E-Prime	DSM-V
Liang et al. (2022)	China	Mean: 6–12 Range: 8.4 ± 1.4	77.50%	80 (40/40)	Cognitive Aerobic	No treatment	60 min ^*^ 3 times ^*^ 12 weeks	ToL Flanker TMT	DSM-V
Nejati and Derakhshan (2021)	Iran	Mean: NA Range: 9.43 ± 1.43	100.00%	30 (15/15)	Cognitive Aerobic	Running	45 min ^*^ 3 times ^*^ 5 weeks	GNG WCST N-Back	DSM-V
Bigelow et al. (2021)	Canada	Mean: 10–14 Range: 11.38 ± 1.5	80.00%	32 (16/16)	Cycling	No treatment	10 min ^*^ 1 times ^*^ 1 weeks	Stroop Leiter-3 TMT	DSM-V
Smith et al. (2020)	USA	Mean: 5–9 Range: 7.4 ± 1.1	70.00%	80 (53/27)	Cognitive Aerobic	Regular treatment	45 min ^*^ 3 times ^*^ 15 weeks	CVLT Flanker	DSM-IV
Geladé et al. (2018)	Netherlands	Mean: 7–13 Range: 9.6 ± 1.67	78.00%	92 (33/28/31)	Simple Aerobic	Neurotherapy/medication	45 min ^*^ 3 times ^*^ 12 weeks	VSWM	DSM-V
Pan et al. (2019)	China	Mean: 7–12 Range: 9.08 ± 1.43	100.00%	30 (15/15)	Bad minton	No treatment	70 min ^*^ 2 times ^*^ 12 weeks	Stroop WCST	DSM-IV
Benzing and Schmidt (2019)	Switzerland	Mean: 8–12 Range: 10.46 ± 1.30	82.40%	51 (28/23)	Exerga ming	No treatment	30 min ^*^ 3 times ^*^ 8 weeks	Simon Flanker	ICD-10
Rezaei et al. (2018)	Iran	Mean: 7–11 Range: NA	NA	14 (7/7)	Yoga	No treatment	45 min ^*^ 3 times ^*^ 8 weeks	CPT WISC-R	DSM-V
Benzing et al. (2018)	Switzerland	Mean: 8–12 Range: 10.46 ± 1.35	82.60%	46 (24/22)	Exerga ming	Watching videos	15 min ^*^ 1 times ^*^ 1 weeks	Stroop FT CSBT	ICD-10
Geladé et al. (2017)	Netherlands	Mean: 7–13 Range: 9.8 ± 2.0	75.90%	112 (37/39/36)	Simple Aerobic	Neurotherapy/medication	45 min ^*^ 3 times ^*^ 12 weeks	SST VSWM	DSM-IV
Bustamante et al. (2016)	USA	Mean: 6–12 Range: 9.4 ± 2.2	69.00%	35 (19/16)	Simple Aerobic	Sedentary	90 min ^*^ 5 times ^*^ 10 weeks	STOPIT AWMA	DSM-IV
Ruiter et al. (2022)	Netherlands	Mean: NA Range: 15.62 ± 2.20	94.40%	18 (18/18)	Cycling	Sedentary	30 min ^*^ 1 times ^*^ 1 weeks	Phonological WM Task	DSM-V
Hung et al. (2016)	China	Mean: 8–12 Range: 10.24 ± 1.78	97.00%	40 (20/20)	Running	Watching videos	30 min ^*^ 1 times ^*^ 1 weeks	TSP	Hospital Certificate
Chang et al. (2012)	China	Mean: 8–13 Range: 10.43	93.00%	40 (20/20)	Running	Watching videos	30 min ^*^ 1 times ^*^ 1 weeks	Stroop WCST	DSM-IV
Ziereis and Jansen (2015)	Germany	Mean: 7–12 Range: 9.2 ± 1.3	74.00%	43 (13/14/16)	Cognitive Aerobic	No treatment	60 min ^*^ 1 times ^*^ 12 weeks	DSFBT LNST	ICD-10

For convenience, only the first author is given. E/C means experimental group/control group; NA, no report; GNG, Go/No-Go Task; N-Back, N-Back Task; E-Prime, E-Prime Software for Psychological Experimentation; ToL, Tower of London; TMT, Trail Making Test; WCST, Wisconsin Card Sorting Test; Stroop, Stroop Task; Leiter-3, Leiter International Performance Scale-Third Edition; CVLT, California Verbal Learning Test; Flanker, Flanker Task; NeuroTrax, NeuroTrax Cognitive Test; CPT, Continuous Performance Test; WISC-R, Wechsler Intelligence Scale for Children - Revised; CSBT, Color Span Backward Task; Auditory Oddball, Auditory Oddball Task; SST, Stop-Signal Task; VSWM, Visual-Spatial Working Memory Task; STOPIT, Stop-Signal Task; AWMA, Automated Working Memory Assessment; Phonological WM, Phonological Working Memory Task; TSP, Task Switching Paradigm; DSFBT, Digit Span Forward and Backward Task; LNST, Letter-Number Sequencing Task.

### 3.1 Quality assessment of the included studies

The quality of the included studies was assessed using the PEDro scale for randomized controlled trials (RCTs) and the MINORS scale for non-randomized controlled trials (non-RCTs). A total of 13 RCTs were evaluated with PEDro scores ranging from 4 to 7, indicating moderate methodological quality. Most studies scored well on criteria such as eligibility criteria, random allocation, concealed allocation, and statistical comparisons of primary outcomes. However, lower scores were observed for items related to baseline similarity, blinding of participants, and blinding of therapists, indicating potential risks of bias in these areas. For the non-RCTs, four studies were assessed using the MINORS scale, with total scores ranging from 14 to 16, suggesting relatively high methodological quality. These studies performed well in areas such as clearly stated aims, appropriate endpoints, and baseline equivalence, while lower scores were noted for the lack of blinded assessments and prospective calculation of sample size. Overall, the quality assessment ensured that only studies with sufficient methodological rigor were included in the analysis, though some risk of bias remains and should be considered in the interpretation of results.

### 3.2 Risk assessment results of literature bias

In this study, we used the RoB 2 and ROBINS-I tools to assess the risk of bias in randomized controlled trials (RCTs) and non-randomized studies, respectively. Among the 13 RCTs, only two studies had a low risk of bias in all evaluation areas. Approximately 60% of the RCTs had some concerns or a high risk of bias. For the four non-randomized studies included, controlling for confounding factors was the main limitation. Two of these studies were rated as high risk. However, the selection of participants and classification of interventions were handled well, both showing a low risk of bias. It is important to note that non-randomized studies also had some limitations in outcome measurement and reporting, with about 50% of these studies showing some issues. Due to the nature of the intervention, it was difficult to implement double-blinding for both participants and researchers. Additionally, monitoring and quantifying exercise adherence was challenging, which may have affected the standardization of the intervention (see Appendix Tables 1, 2).

### 3.3 Results of data analysis

The network meta-analysis assessed the impact of various exercise interventions on working memory in children with ADHD. The results indicated a moderate effect of exercise interventions on working memory. To validate the robustness of the analysis, an inconsistency network model was employed to examine the overall consistency between both pairwise and multi-arm direct and indirect effects simultaneously. Furthermore, model convergence was evaluated using the potential scale reduction factor (PSRF), with results approaching 1, indicating robust convergence (see Appendix Figures 1, 2).

The findings showed minimal differences in the deviance information criterion (DIC) between the consistency model and the inconsistency model, with a difference of < 5, suggesting no significant disparity between the two models, and no evident inconsistency (see Appendix Figure 3). The loop inconsistency model was used to assess the A-B-D loop, revealing an inconsistency factor (IF) of 0.036, standard error (seIF) of 0.517, a z-value of 0.070, and a *p*-value of 0.944, with a 95% confidence interval of (0.00, 1.05). Since the *p*-value was > 0.05 and the confidence interval included 0, it suggests no significant inconsistency between direct and indirect effects within the loop. Moreover, the heterogeneity within the loop was 0.064, indicating low heterogeneity, further supporting the consistency of direct and indirect evidence within the loop (see Appendix Figure 4). The adjusted funnel plot showed no significant publication bias (see Appendix Figure 5).

#### 3.3.1 The impact of sports activities on working memory in children with ADHD

**Figure 2:**
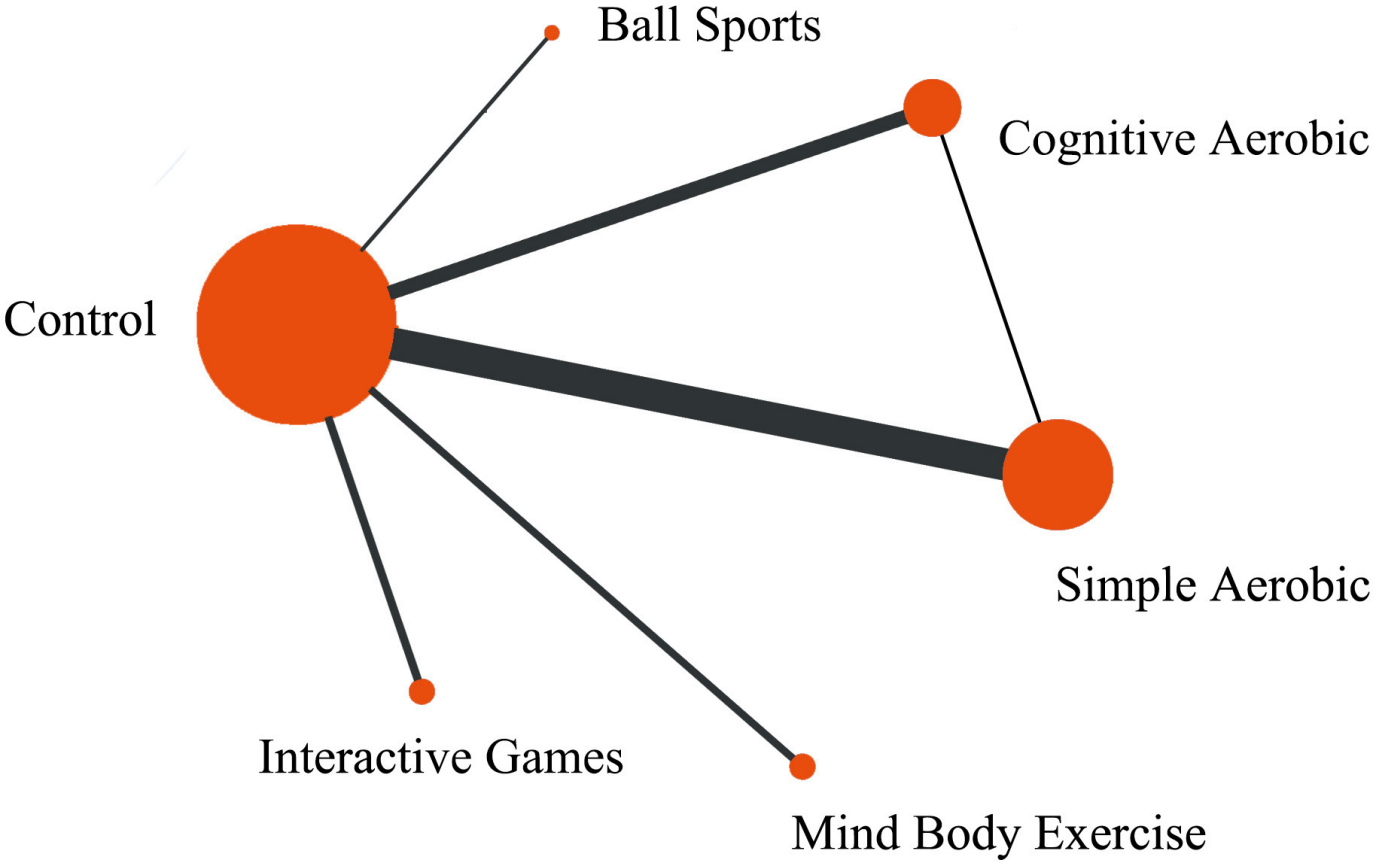
Comparison and efficacy network diagram of different treatments on memory dimensions compared to the control group.

The network plot displays the comparative relationships between different exercise interventions (such as ball sports, cognitive-aerobic exercise, simple aerobic exercise, mind-body exercise, and interactive games) and the control group. The size of the nodes reflects the sample size, with the control group, cognitive-aerobic exercise, and simple aerobic exercise having the largest sample sizes. The thickness of the connecting lines represents the abundance of comparison data, with thicker lines between the control group and cognitive-aerobic exercise, and simple aerobic exercise, indicating more extensive data for these interventions. Comparatively, the data for ball sports, interactive games, and mind-body exercise are less abundant. The plot shows fewer closed loops, suggesting that direct comparisons among interventions are limited, and the majority of the studies focus on comparisons between the interventions and the control group (see Figure 2).

**Figure 3:**
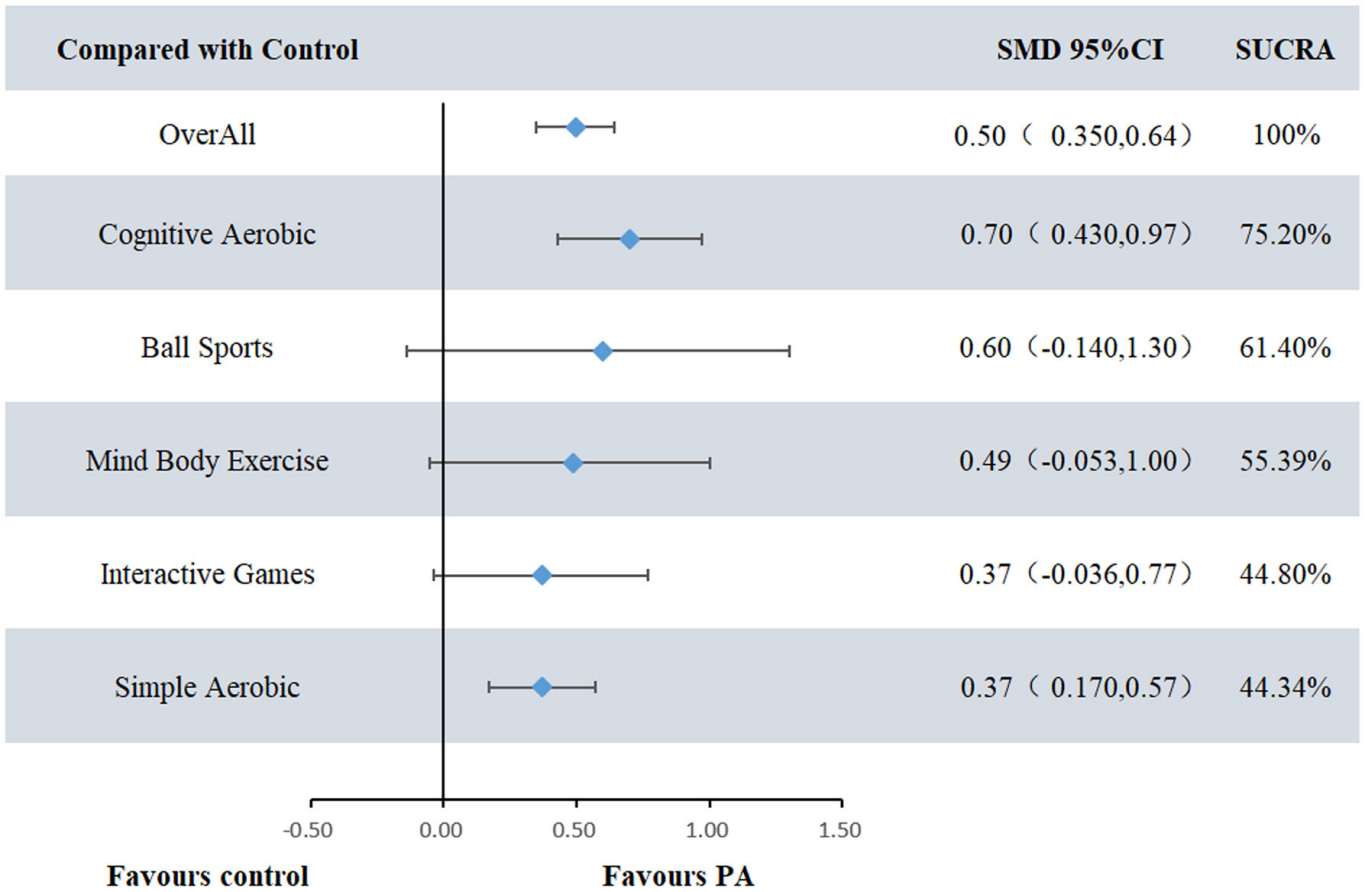
Forest chart, PA, physical activity, standard mean deviation, SUCRA, cumulative ranking curve beneath the surface.

**Figure 4:**
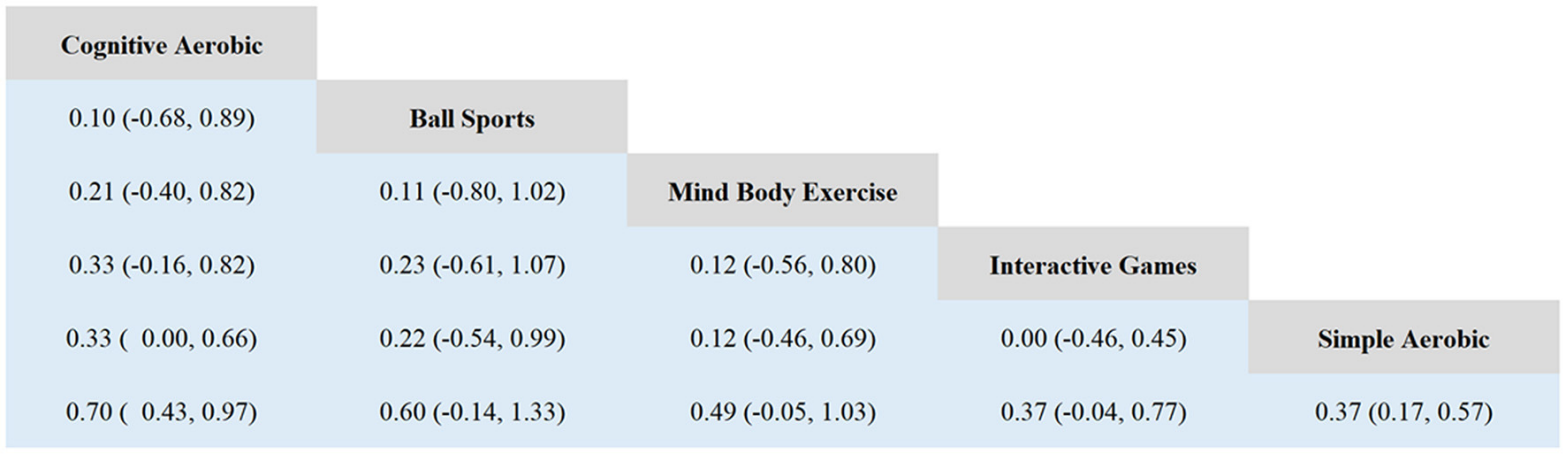
League table.

Based on the forest plot and SUCRA ranking analysis, significant differences were observed in the effectiveness of various exercise interventions in improving working memory in children with ADHD. The network meta-analysis revealed a moderate overall effect size (SMD = 0.50, 95% CI: 0.35–0.64), with cognitive-aerobic exercise demonstrating the most significant effect (SMD = 0.72, 95% CI: 0.44–1.00) and the highest SUCRA value (75.21%). Ball sports ranked second with a moderate to high improvement effect (SMD = 0.61, 95% CI: −0.12–1.35) and a SUCRA value of 61.28%. Mind-body exercises (SMD = 0.50, 95% CI: −0.04–1.04, SUCRA = 55.46%) and interactive games (SMD = 0.37, 95% CI: −0.03–0.78, SUCRA = 44.91%) showed moderate effects. Simple aerobic exercise exhibited a smaller improvement effect (SMD = 0.40, 95% CI: 0.19–0.60) with a SUCRA value of 44.28%, while the control group showed minimal improvement (SUCRA = 10.53%). These results highlight the superior effectiveness of cognitive-aerobic exercise in enhancing working memory in children with ADHD, followed by ball sports and mind-body exercises (see Figures 3, 4).

To explore potential moderators of the intervention effects, a meta-regression analysis was conducted, incorporating variables such as the mean age of participants, gender ratio, weekly intervention frequency, total intervention duration, session duration, and overall intervention period. The results indicated that none of these factors significantly moderated the effect of exercise interventions, further confirming the robustness of the findings (see Appendix Figure 6).

In this study, the meta-regression analysis aimed to examine potential moderators influencing the exercise intervention effects by including the following variables in the model: year of publication (the time the study was conducted), participant age (age distribution of children with ADHD), gender ratio (gender composition), weekly intervention frequency (the number of interventions per week), total intervention time (overall duration of the intervention), session duration (duration of each intervention), and total intervention period (the overall duration of the intervention program). The purpose of incorporating these variables was to assess how different experimental designs and population characteristics might moderate the effects of exercise interventions, thereby confirming the robustness of the study's results.

The analysis revealed that, with the exception of weekly intervention frequency and total intervention time, which significantly influenced the effects of cognitive-aerobic exercise, the other variables (including year of publication, participant age, gender ratio, session duration, and total intervention period) did not significantly moderate the effect of exercise interventions. Specifically, the results showed that increased weekly intervention frequency and total intervention time significantly enhanced the effect of cognitive-aerobic exercise in improving working memory in children with ADHD. This suggests that the effectiveness of cognitive-aerobic exercise may depend on higher intervention frequency and longer cumulative intervention duration.

In summary, cognitive-aerobic exercise and ball sports were found to be the most effective in improving working memory in children with ADHD, while the effect of simple aerobic exercise was more limited. The network meta-analysis integrated both direct and indirect evidence, ensuring the robustness of the results. By combining effect sizes with cumulative ranking values (SUCRA), researchers can gain a clearer understanding of the strength and relative priority of different interventions. However, some interventions exhibited wider confidence intervals for effect sizes, suggesting that the results may be influenced by sample size and heterogeneity of the interventions. Future studies could consider increasing sample sizes and improving intervention designs to enhance the precision and consistency of the findings. Additionally, sensitivity analyses and publication bias tests indicated that the results of this study were relatively robust, providing support for the reliability of the intervention effects.

## 4 Discussion

**Figure 5:**
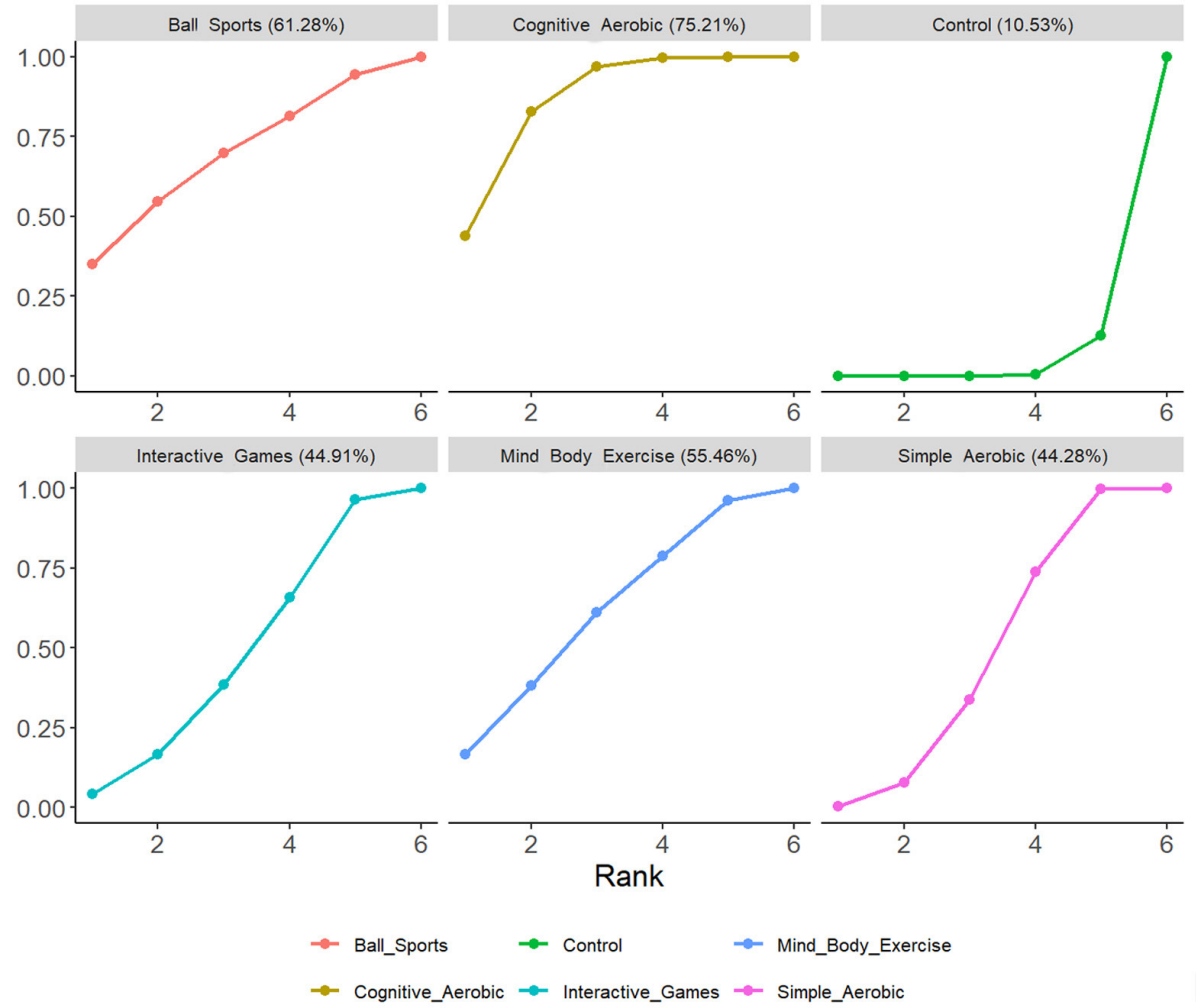
Cumulative probability.

Based on the cumulative probability ranking and coverage area shown in Figure 5, significant differences exist in the effects of various types of exercise interventions on improving working memory in children with ADHD. In the SUCRA cumulative probability analysis, a larger coverage area indicates a higher rank in terms of intervention effectiveness, meaning the intervention has a stronger effect on enhancing working memory. From the figure, it can be observed that cognitive-aerobic exercise has the largest coverage area at 75.21%, indicating that this intervention is the most effective among all types. This larger coverage area suggests that cognitive-aerobic exercise has a clear advantage in improving working memory in children with ADHD. Following closely is ball sports, with a coverage area of 61.28%, also showing a good improvement effect. Mind-body exercises have a coverage area of 55.46%, ranking third, indicating a moderate improvement effect. Interactive games and simple aerobic exercises have relatively smaller coverage areas, at 44.91% and 44.28%, respectively, suggesting that their effects on improving working memory are more moderate. Finally, the control group has the smallest coverage area, at only 10.53%, showing almost no improvement.

These differences in coverage areas reflect the varying cognitive load, executive demands, and complexity involved in each type of exercise intervention. The disparity in results may stem from the distinct cognitive demands of different exercise types on children with ADHD and the degree to which they activate executive functions. Cognitive-aerobic exercise shows the most significant effect (SMD = 0.72), which may be because this type of exercise combines the dual demands of aerobic activity and cognitive tasks, requiring children to continuously process information, maintain focus, and perform complex cognitive operations during the exercise (Yu et al., 2018; McDaniel et al., 2014). Cognitive-aerobic exercise may involve tasks with rules and objectives, which increase the load on the prefrontal cortex, making it highly active in multitasking situations (Koch et al., 2020). The prefrontal cortex is closely associated with working memory, and this sustained cognitive stimulation helps improve working memory function (Curtis and D'Esposito, 2003). Furthermore, cognitive-aerobic exercise may involve repeated cognitive switching, decision-making, and memory retrieval, which effectively trains children's ability to retain and update information, thus showing significant effects in enhancing working memory (Soltani Kouhbanani and Rothenberger, 2021).

Ball sports rank second in effectiveness (SMD = 0.61), and their positive impact on working memory may stem from the strategic and team-oriented demands of these sports. For example, sports such as soccer or basketball typically require children to remain highly focused in dynamic and competitive situations, while also remembering and analyzing the actions of teammates and opponents, which places a high demand on task memory. In ball sports, children not only need to plan and execute movements but also continuously adjust strategies and predict the opponent's actions. These multitasking and real-time adjustment characteristics directly exercise their working memory load, information storage, and response speed (Ishihara and Mizuno, 2018; Ishihara et al., 2017).

In contrast, mind-body exercises (SMD = 0.50) and interactive games (SMD = 0.37) show moderate effects. This is likely because these activities may be more significant in improving attention and emotional regulation but present relatively lower direct challenges to executive functions and cognitive load (Wang et al., 2023). Mind-body exercises such as yoga and Tai Chi typically emphasize concentration, breath control, and relaxation. Studies have shown that such exercises help reduce anxiety levels in children with ADHD and enhance the stability of their attention (Wu et al., 2013; Rao et al., 2019). While these effects indirectly improve working memory, they may lack the high cognitive load stimulation required for direct improvements. Virtual sports-based interactive games primarily focus on enhancing children's social interaction and cooperation experiences, such as motivating participants through collaborative tasks or fun competitions in virtual environments. Although these games have a positive impact on the social behavior and emotional regulation of children with ADHD (Ochi et al., 2024; Zhao et al., 2022), their lower task complexity and memory load requirements mean they are less effective than cognitive-aerobic exercise and ball sports in directly improving working memory. The cognitive challenges in these games are limited and generally do not involve high-intensity memory tasks or complex decision-making, making their direct impact on working memory relatively modest.

On the other hand, the effect of regular aerobic exercise is the weakest (SMD = 0.40), which may be attributed to its simpler exercise format and lower cognitive load. Activities like running and skipping, while improving overall physical fitness and stimulating dopamine secretion, can help children with ADHD maintain attention in the short term (Kuo et al., 2024; Ren et al., 2024). However, since they lack demands for memory and multitasking, they are often insufficient to activate the prefrontal cortex's executive function areas. As a result, their direct impact on working memory is relatively small.

Compared to existing studies, this network meta-analysis is the first to systematically evaluate the effects of different exercise interventions on working memory in children with ADHD. Our findings revealed that cognitive-aerobic exercise showed the most significant improvement (SMD = 0.72, 95% CI: 0.44–1.00). This effect size is notably larger than that reported in a recent meta-analysis of pure aerobic exercise (SMD = 0.48, 95% CI: 0.02–0.95). The difference suggests a potential synergistic effect when cognitive tasks are integrated into aerobic exercise. Our study identified intervention frequency and total duration as key factors affecting intervention outcomes. This aligns with Yang et al. (2024)'s findings, which emphasized the effectiveness of exercise programs lasting 6–12 weeks with 60–90 min per session. Previous research has primarily focused on single types of exercise. For instance, Sun et al. (2024) only examined structured physical exercise. In contrast, our systematic comparison of various exercise types provides more comprehensive guidance for clinical practice. Notably, our results indicate that ball games may have considerable beneficial effects (SMD = 0.61). This finding has received limited attention in previous research. It opens new directions for future studies in this field.

In summary, these differences may reflect variations in cognitive load, executive demands, and the degree of prefrontal cortex activation across different types of exercise interventions, leading to significantly different effects on the improvement of working memory in children with ADHD. Cognitive-aerobic exercise and ball sports, due to their higher cognitive demands and dynamic complexity, have been more effective in promoting improvements in working memory (Jacobson and Matthaeus, 2014). In contrast, mind-body exercises and interactive games, which emphasize emotional regulation and simple social tasks, show moderate effects. Regular aerobic exercise, due to its more straightforward physical activity format, has the smallest impact on working memory improvement. These findings suggest that when designing exercise interventions for children with ADHD, priority should be given to exercise types with higher cognitive load in order to more effectively enhance working memory.

Although this study reveals the differences in the effectiveness of various exercise interventions in improving working memory in children with ADHD, several limitations remain. For instance, the underlying mechanisms of these interventions have not been thoroughly explored, and there is a lack of long-term follow-up data. Additionally, there is considerable heterogeneity among the samples, and children of different ages or with varying levels of symptom severity may respond differently to the interventions. These findings have important theoretical and practical implications. Future research should further explore the specific mechanisms by which different types of exercise affect working memory, such as through neuroimaging techniques to understand the advantages of cognitive-aerobic exercise and ball sports. For practical implementation, we recommend integrating structured physical activities into daily classroom routines, such as incorporating cognitive-motor exercises between lessons or using active learning strategies that combine physical movement with cognitive tasks. School administrators should consider providing professional development opportunities for teachers to effectively implement these interventions, while clinicians could incorporate combined cognitive-physical exercises into treatment protocols with individualized intensity based on symptom severity. Future studies should conduct individualized analyses to explore how different subgroups respond to various exercise interventions, assess the long-term sustainability of intervention effects, and test their feasibility in real classroom settings. Additionally, optimizing the frequency, intensity, and duration of interventions through interdisciplinary collaboration would help maximize improvements in working memory. These efforts would collectively advance both the theoretical understanding and practical application of exercise interventions for children with ADHD.

## 5 Conclusion

Concluding, this study used network meta-analysis to investigate the effects of different types of exercise interventions on working memory in children with ADHD. The results indicate that cognitive-aerobic exercise and ball sports are significantly more effective than other types of exercise interventions in improving working memory. This difference may be attributed to the varying cognitive load, task complexity, and the degree of activation of executive functions across different exercise types. The findings suggest that when designing exercise interventions for children with ADHD, priority should be given to exercise types with higher cognitive load in order to more effectively enhance working memory.

## Data Availability

The original contributions presented in the study are included in the article/Supplementary material, further inquiries can be directed to the corresponding author.
