# Supplementary material for: Exploring the impact of different types of exercise on working memory in children with ADHD: a network meta-analysis
Source: Front Psychol. 2025 Jan 27;16:1522944. doi: 10.3389/fpsyg.2025.1522944 (PMC11808027; doi:10.3389/fpsyg.2025.1522944)
Supplement: Supplementary file 1 [file Data_Sheet_1.pdf]

Appendix

Supporting Documentation

Search strategy

Figure 1 Convergence diagnosis

Figure 2 Trajectory and density plots for consistency model testing of various intervention measures

Figure 3 Consistency model and inconsistency model

Figure 4 Cumulative Probability

Figure 5 Funnel plots of publication bias for outcome measures of included studies

Figure 6-7 Regressive analysis

Figure 8-9 RoB2 adn ROBINS-I V2 literature publication bias

Table 1 MINORS literature quality evaluation table

Table 2 PEDro literature Quality evaluation

PRISMA NMA Checklist of Items to Include When Reporting A Systematic Review Involving a Network Meta-analysis.

| Section/Topic | Item # | Checklist Item | Reported on Page # |
|---|---|---|---|
| **TITLE** | | | |
| Title | 1 | Identify the report as a systematic review *incorporating a network meta-analysis (or related form of meta-analysis).* | |
| **ABSTRACT** | | | |
| Structured summary | 2 | Provide a structured summary including, as applicable:<br>**Background:** main objectives<br>**Methods:** data sources; study eligibility criteria, participants, and interventions; study appraisal; and *synthesis methods, such as network meta-analysis.*<br>**Results:** number of studies and participants identified; summary estimates with corresponding confidence/credible intervals; *treatment rankings may also be discussed. Authors may choose to summarize pairwise comparisons against a chosen treatment included in their analyses for brevity.*<br>**Discussion/Conclusions:** limitations; conclusions and implications of findings.<br>**Other:** primary source of funding; systematic review registration number with registry name. | |
| **INTRODUCTION** | | | |
| Rationale | 3 | Describe the rationale for the review in the context of what is already known, *including mention of why a network meta-analysis has been conducted.* | |
| Objectives | 4 | Provide an explicit statement of questions being addressed, with reference to participants, interventions, comparisons, outcomes, and study design (PICOS). | |
| **METHODS** | | | |
| Protocol and registration | 5 | Indicate whether a review protocol exists and if and where it can be accessed (e.g., Web address); and, if available, provide registration information, including registration number. | |
| Eligibility criteria | 6 | Specify study characteristics (e.g., PICOS, length of follow-up) and report characteristics (e.g., years considered, language, publication status) used as criteria for eligibility, giving rationale. *Clearly describe eligible treatments included in the treatment network, and note whether any have been clustered or merged into the same node (with justification).* | |
| Information sources | 7 | Describe all information sources (e.g., databases with dates of coverage, contact with study authors to identify additional studies) in the search and date last searched. | |
| Search | 8 | Present full electronic search strategy for at least one database, including any limits used, such that it could be repeated. | |
| Study selection | 9 | State the process for selecting studies (i.e., screening, eligibility, included in systematic review, and, if applicable, | |

| | | | |
|---|---|---|---|
| | | included in the meta-analysis). | |
| Data collection process | 10 | Describe method of data extraction from reports (e.g., piloted forms, independently, in duplicate) and any processes for obtaining and confirming data from investigators. | |
| Data items | 11 | List and define all variables for which data were sought (e.g., PICOS, funding sources) and any assumptions and simplifications made. | |
| **Geometry of the network** | **S1** | Describe methods used to explore the geometry of the treatment network under study and potential biases related to it. This should include how the evidence base has been graphically summarized for presentation, and what characteristics were compiled and used to describe the evidence base to readers. | |
| Risk of bias within individual studies | 12 | Describe methods used for assessing risk of bias of individual studies (including specification of whether this was done at the study or outcome level), and how this information is to be used in any data synthesis. | |
| Summary measures | 13 | State the principal summary measures (e.g., risk ratio, difference in means). *Also describe the use of additional summary measures assessed, such as treatment rankings and surface under the cumulative ranking curve (SUCRA) values, as well as modified approaches used to present summary findings from meta-analyses.* | |
| Planned methods of analysis | 14 | Describe the methods of handling data and combining results of studies for each network meta-analysis. This should include, but not be limited to:<br>• *Handling of multi-arm trials;*<br>• *Selection of variance structure;*<br>• *Selection of prior distributions in Bayesian analyses; and*<br>• *Assessment of model fit.* | |
| **Assessment of Inconsistency** | **S2** | Describe the statistical methods used to evaluate the agreement of direct and indirect evidence in the treatment network(s) studied. Describe efforts taken to address its presence when found. | |
| Risk of bias across studies | 15 | Specify any assessment of risk of bias that may affect the cumulative evidence (e.g., publication bias, selective reporting within studies). | |
| Additional analyses | 16 | Describe methods of additional analyses if done, indicating which were pre-specified. This may include, but not be limited to, the following:<br>• Sensitivity or subgroup analyses;<br>• Meta-regression analyses;<br>• *Alternative formulations of the treatment network; and*<br>• *Use of alternative prior distributions for Bayesian analyses (if applicable).* | |

**RESULTS†**

| | | | |
|---|---|---|---|
| Study selection | 17 | Give numbers of studies screened, assessed for eligibility, and included in the review, with reasons for exclusions at each stage, ideally with a flow diagram. | |
| **Presentation of network structure** | **S3** | Provide a network graph of the included studies to enable visualization of the geometry of the treatment network. | |
| **Summary of network geometry** | **S4** | Provide a brief overview of characteristics of the treatment network. This may include commentary on the abundance of trials and randomized patients for the different interventions and pairwise comparisons in the network, gaps of evidence in the treatment network, and potential biases reflected by the network structure. | |
| Study characteristics | 18 | For each study, present characteristics for which data were extracted (e.g., study size, PICOS, follow-up period) and provide the citations. | |
| Risk of bias within studies | 19 | Present data on risk of bias of each study and, if available, any outcome level assessment. | |
| Results of individual studies | 20 | For all outcomes considered (benefits or harms), present, for each study: 1) simple summary data for each intervention group, and 2) effect estimates and confidence intervals. *Modified approaches may be needed to deal with information from larger networks.* | |
| Synthesis of results | 21 | Present results of each meta-analysis done, including confidence/credible intervals. *In larger networks, authors may focus on comparisons versus a particular comparator (e.g. placebo or standard care), with full findings presented in an appendix. League tables and forest plots may be considered to summarize pairwise comparisons.* If additional summary measures were explored (such as treatment rankings), these should also be presented. | |
| **Exploration for inconsistency** | **S5** | Describe results from investigations of inconsistency. This may include such information as measures of model fit to compare consistency and inconsistency models, *P* values from statistical tests, or summary of inconsistency estimates from different parts of the treatment network. | |
| Risk of bias across studies | 22 | Present results of any assessment of risk of bias across studies for the evidence base being studied. | |
| Results of additional analyses | 23 | Give results of additional analyses, if done (e.g., sensitivity or subgroup analyses, meta-regression analyses, *alternative network geometries studied, alternative choice of prior distributions for Bayesian analyses,* and so forth). | |

**DISCUSSION**

| | | | |
|---|---|---|---|
| Summary of evidence | 24 | Summarize the main findings, including the strength of evidence for each main outcome; consider their relevance to key groups (e.g., healthcare providers, users, and policy-makers). | |
| Limitations | 25 | Discuss limitations at study and outcome level (e.g., risk of bias), and at review level (e.g., incomplete retrieval of identified research, reporting bias). *Comment on the validity of the assumptions, such as transitivity and consistency. Comment* | |

| | | | |
|---|---|---|---|
| | | *on any concerns regarding network geometry (e.g., avoidance of certain comparisons).* | |
| Conclusions | 26 | Provide a general interpretation of the results in the context of other evidence, and implications for future research. | |
| **FUNDING** | | | |
| Funding | 27 | Describe sources of funding for the systematic review and other support (e.g., supply of data); role of funders for the systematic review. This should also include information regarding whether funding has been received from manufacturers of treatments in the network and/or whether some of the authors are content experts with professional conflicts of interest that could affect use of treatments in the network. | |

PICOS = population, intervention, comparators, outcomes, study design.
* Text in italics indicateS wording specific to reporting of network meta-analyses that has been added to guidance from the PRISMA statement.
† Authors may wish to plan for use of appendices to present all relevant information in full detail for items in this section.

Search strategy

PubMed：1113

#1：((((((((((((((((((((((Attention Deficit Disorders with Hyperactivity[MeSH Terms]) OR (Attention Deficit Disorders with Hyperactivity[Title/Abstract])) OR (ADHD[Title/Abstract])) OR (Attention Deficit Hyperactivity Disorder[Title/Abstract])) OR (Hyperkinetic Syndrome[Title/Abstract])) OR (Syndromes, Hyperkinetic[Title/Abstract])) OR (Attention Deficit-Hyperactivity Disorder[Title/Abstract])) OR (Attention Deficit-Hyperactivity Disorders[Title/Abstract])) OR (Deficit-Hyperactivity Disorder, Attention[Title/Abstract])) OR (Deficit-Hyperactivity Disorders, Attention[Title/Abstract])) OR (Disorder, Attention Deficit-Hyperactivity[Title/Abstract])) OR (Disorders, Attention Deficit-Hyperactivity[Title/Abstract])) OR (ADDH[Title/Abstract])) OR (Attention Deficit Hyperactivity Disorders[Title/Abstract])) OR (Attention Deficit Disorder[Title/Abstract])) OR (Attention Deficit Disorders[Title/Abstract])) OR (Deficit Disorder, Attention[Title/Abstract])) OR (Deficit Disorders, Attention[Title/Abstract])) OR (Disorder, Attention Deficit[Title/Abstract])) OR (Disorders, Attention Deficit[Title/Abstract])) OR (Brain Dysfunction, Minimal[Title/Abstract])) OR (Dysfunction, Minimal Brain[Title/Abstract])) OR (Minimal Brain Dysfunction[Title/Abstract])

#2：((((Sports[MeSH Terms]) OR (((Sport[Title/Abstract]) OR (Athletics[Title/Abstract])) OR (Athletic[Title/Abstract]))) OR ((Exercise Therapy[MeSH Terms]) OR (((((((((((Remedial Exercise[Title/Abstract]) OR (Exercise, Remedial[Title/Abstract])) OR (Exercises, Remedial[Title/Abstract])) OR (Remedial Exercises[Title/Abstract])) OR (Therapy, Exercise[Title/Abstract])) OR (Exercise Therapies[Title/Abstract])) OR (Therapies, Exercise[Title/Abstract])) OR (Rehabilitation Exercise[Title/Abstract])) OR (Exercise, Rehabilitation[Title/Abstract])) OR (Exercises, Rehabilitation[Title/Abstract])) OR (Rehabilitation Exercises[Title/Abstract])))) OR ((Exercise[MeSH Terms]) OR ((((((((((((((((((((((((Exercises[Title/Abstract]) OR (Physical Activity[Title/Abstract])) OR (Activities, Physical[Title/Abstract])) OR (Activity, Physical[Title/Abstract])) OR (Physical Activities[Title/Abstract])) OR (Exercise, Physical[Title/Abstract])) OR (Exercises, Physical[Title/Abstract])) OR (Physical Exercise[Title/Abstract])) OR (Physical Exercises[Title/Abstract])) OR (Acute Exercise[Title/Abstract])) OR (Acute Exercises[Title/Abstract])) OR (Exercise, Acute[Title/Abstract])) OR (Exercises, Acute[Title/Abstract])) OR (Exercise, Isometric[Title/Abstract])) OR (Exercises, Isometric[Title/Abstract])) OR (Isometric Exercises[Title/Abstract])) OR (Isometric Exercise[Title/Abstract])) OR (Exercise, Aerobic[Title/Abstract])) OR (Exercise, Aerobic[Title/Abstract])) OR (Aerobic Exercise[Title/Abstract])) OR (Aerobic Exercises[Title/Abstract])) OR (Exercises, Aerobic[Title/Abstract])) OR (Exercise Training[Title/Abstract])) OR (Trainings, Exercise[Title/Abstract])))) OR (((((((((((((((((((((((((((((((((((((((((((Baseball[Title/Abstract]) OR (Basketball[Title/Abstract])) OR (Bicycling[Title/Abstract])) OR (Boxing[Title/Abstract])) OR (Cricket Sport[Title/Abstract])) OR (Football[Title/Abstract])) OR (Golf[Title/Abstract])) OR (Gymnastics[Title/Abstract])) OR (Hockey[Title/Abstract])) OR (Martial Arts[Title/Abstract])) OR (Mountaineering[Title/Abstract])) OR

(Racquet[Title/Abstract])) OR (Return[Title/Abstract])) OR (Sport[Title/Abstract])) OR (Sports[Title/Abstract])) OR (Rugby[Title/Abstract])) OR (Running[Title/Abstract])) OR (Skating[Title/Abstract])) OR (Snow Sports[Title/Abstract])) OR (Soccer[Title/Abstract])) OR (train[Title/Abstract])) OR (fitness[Title/Abstract])) OR (aerobic[Title/Abstract])) OR (walking[Title/Abstract])) OR (high intensity interval[Title/Abstract])) OR (resistance[Title/Abstract])) OR (core stability[Title/Abstract])) OR (dance[Title/Abstract])) OR (breathing exercise[Title/Abstract])) OR (virtual reality exercise[Title/Abstract])) OR (whole body vibration exercise[Title/Abstract])) OR (stretching[Title/Abstract])) OR (body - mind exercise[Title/Abstract])) OR (Yoga[Title/Abstract])) OR (pilates[Title/Abstract])) OR (Tai Chi[Title/Abstract])) OR (Taijiquan[Title/Abstract])) OR (Health Qigong[Title/Abstract])) OR (Yijinjing[Title/Abstract])) OR (Wuqinxi[Title/Abstract])) OR (Liuzijue[Title/Abstract])) OR (Baduanjin[Title/Abstract])) OR (multicomponent exercise[Title/Abstract]))

#3：(((((((((((((((((((((((Child[MeSH Terms]) OR (Adolescent[MeSH Terms])) OR (Young Adult[MeSH Terms])) OR (Minor[MeSH Terms])) OR (Adolescen[Title/Abstract])) OR (Teen[Title/Abstract])) OR (Teenager[Title/Abstract])) OR (Youth[Title/Abstract])) OR (Minors[Title/Abstract])) OR (Minor[Title/Abstract])) OR (Child[Title/Abstract])) OR (Child[Title/Abstract])) OR (Kid[Title/Abstract])) OR (Kids[Title/Abstract])) OR (Girl[Title/Abstract])) OR (Boy[Title/Abstract])) OR (Under age[Title/Abstract])) OR (Underage[Title/Abstract])) OR (Young people[Title/Abstract])) OR (young person[Title/Abstract])) OR (Pubescen[Title/Abstract])) OR (Young Adult[Title/Abstract])) OR (School age[Title/Abstract])) OR (Preschool[Title/Abstract])) OR (Student[Title/Abstract])

#4：#1 AND #2 AND #3

WOS：4839

#1：TS=("Attention Deficit Hyperactivity Disorder" OR "ADHD" OR "Attention-Deficit/Hyperactivity Disorder")

#2：(TS=(Equine-Assisted Therapy)) OR (TS=(Animal Assisted Therapy)) OR (TS=(Baseball)) OR (TS=(Basketball)) OR (TS=(Bicycling)) OR (TS=(Boxing)) OR (TS=(Cricket Sport)) OR (TS=(Football)) OR (TS=(Golf)) OR (TS=(Gymnastics)) OR (TS=(Hockey)) OR (TS=(Martial Arts)) OR (TS=(Mountaineering)) OR (TS=(Racquet Sports)) OR (TS=(Return to Sport)) OR (TS=(Rugby)) OR (TS=(Running)) OR (TS=(Skating)) OR (TS=(Snow Sports)) OR (TS=(Soccer)) OR (TS=(train)) OR (TS=(fitness)) OR (TS=(aerobic)) OR (TS=(walking)) OR (TS=(high intensity interval)) OR (TS=(resistance)) OR (TS=(core stability)) OR (TS=(dance)) OR (TS=(breathing exercise)) OR (TS=(virtual reality exercise)) OR (TS=(whole body vibration exercise)) OR (TS=(stretching)) OR (TS=(body - mind exercise)) OR (TS=(Yoga)) OR (TS=(pilates)) OR (TS=(Tai Chi)) OR (TS=(Taijiquan)) OR (TS=(Health Qigong)) OR (TS=(Yijinjing)) OR (TS=(Wuqinxi)) OR (TS=(Liuzijue)) OR (TS=(Baduanjin)) OR (TS=(multicomponent exercise)) OR (((TS=(Sports)) OR TS=(Sport)) OR TS=(Athletics)) OR TS=(Athletic)OR ((((((((((TS=(Exercise Therapy)) OR TS=(Remedial Exercise)) OR TS=(Exercise, Remedial)) OR TS=(Exercises, Remedial)) OR TS=(Remedial Exercises)) OR TS=(Therapy, Exercise)) OR TS=(Exercise Therapies)) OR TS=(Therapies, Exercise)) OR TS=(Rehabilitation Exercise)) OR TS=(Exercise, Rehabilitation)) OR TS=(Exercises, Rehabilitation)) OR TS=(Rehabilitation Exercises)

OR ((((((((((((((((((((((((TS=(Exercise)) OR TS=(Exercises)) OR TS=(Physical Activity)) OR TS=(Activities, Physical)) OR TS=(Activity, Physical)) OR TS=(Physical Activities)) OR TS=(Exercise, Physical)) OR TS=(Exercises, Physical)) OR TS=(Physical Exercise)) OR TS=(Physical Exercises)) OR TS=( Acute Exercise)) OR TS=(Acute Exercises)) OR TS=(Exercise, Acute)) OR TS=(Exercises, Acute)) OR TS=(Exercise, Isometric)) OR TS=(Exercises, Isometric)) OR TS=(Isometric Exercises)) OR TS=(Isometric Exercise)) OR TS=(Exercise, Aerobic)) OR TS=(Aerobic Exercise)) OR TS=(Aerobic Exercises)) OR TS=(Exercises, Aerobic)) OR TS=(Exercise Training)) OR TS=(Exercise Trainings)) OR TS=(Training, Exercise)) OR TS=(Trainings, Exercise)

#3：((((((((((((((((((((((((TS=(Child)) OR TS=(Adolescent)) OR TS=(Young Adult )) OR TS=(Minor )) OR TS=(Adolescen)) OR TS=(Teen)) OR TS=(Teenager)) OR TS=(Youth)) OR TS=(Minors)) OR TS=(Minor)) OR TS=(Child)) OR TS=(Kid)) OR TS=(Kids)) OR TS=(Girl)) OR TS=(Boy)) OR TS=(Under age)) OR TS=(Underage)) OR TS=(Young people )) OR TS=(young person )) OR TS=(Prepubescen)) OR TS=(Pubescen)) OR TS=(Young Adult)) OR TS=(School age)) OR TS=(Preschool)) OR TS=(Student)

#4：#1 AND #2 AND #3

Cochrane （607）

#1: Attention Deficit Hyperactivity Disorder

#2: (Attention Deficit Hyperactivity Disorder):ab,ti,kw OR (Attention Deficit Hyperactivity Disorder):ab,ti,kw OR (ADHD):ab,ti,kw OR (Disorder, Attention Deficit Hyperactivity):ab,ti,kw

#3：Therapeutics

#4：(Therapeutic ):ab,ti,kw OR (Therapy ):ab,ti,kw OR (Therapies ):ab,ti,kw OR (Treatment ):ab,ti,kw OR (Treatments ):ab,ti,kw

#5：Sports

#6：(Sport ):ab,ti,kw OR (Athletics ):ab,ti,kw OR (Athletic ):ab,ti,kw

#7：Exercise

#8：(Exercises ):ab,ti,kw OR (Physical Activity ):ab,ti,kw OR (Activities, Physical ):ab,ti,kw OR (Activity, Physical ):ab,ti,kw OR (Physical Activities ):ab,ti,kw OR (Exercise, Physical ):ab,ti,kw OR (Exercises, Physical ):ab,ti,kw OR (Physical Exercise ):ab,ti,kw OR (Physical Exercises ):ab,ti,kw OR (Acute Exercise ):ab,ti,kw OR (Acute Exercises ):ab,ti,kw OR (Exercise, Acute ):ab,ti,kw OR (Exercises, Acute ):ab,ti,kw OR (Exercise, Isometric ):ab,ti,kw OR (Exercises, Isometric ):ab,ti,kw OR (Isometric Exercises ):ab,ti,kw OR (Isometric Exercise ):ab,ti,kw OR (Exercise, Aerobic ):ab,ti,kw OR (Aerobic Exercise ):ab,ti,kw OR (Aerobic Exercises ):ab,ti,kw OR (Exercises, Aerobic ):ab,ti,kw OR (Exercise Training ):ab,ti,kw OR (Exercise Trainings ):ab,ti,kw OR (Training, Exercise ):ab,ti,kw OR (Trainings, Exercise ):ab,ti,kw

#9：(Baseball ):ab,ti,kw OR (Basketball ):ab,ti,kw OR (Bicycling ):ab,ti,kw OR (Boxing ):ab,ti,kw OR (Cricket Sport ):ab,ti,kw OR (Football ):ab,ti,kw OR (Golf ):ab,ti,kw OR (Gymnastics ):ab,ti,kw OR (Hockey ):ab,ti,kw OR (Martial Arts ):ab,ti,kw OR (Mountaineering ):ab,ti,kw OR (Racquet Sports ):ab,ti,kw OR (Return to Sport ):ab,ti,kw OR (Rugby ):ab,ti,kw OR (Running ):ab,ti,kw OR (Skating ):ab,ti,kw OR (Snow Sports ):ab,ti,kw OR (Soccer ):ab,ti,kw OR (train ):ab,ti,kw OR (fitness ):ab,ti,kw OR (aerobic ):ab,ti,kw OR (walking ):ab,ti,kw OR (high intensity interval ):ab,ti,kw OR (resistance ):ab,ti,kw OR (core stability ):ab,ti,kw OR (dance ):ab,ti,kw OR (breathing exercise ):ab,ti,kw OR (virtual reality exercise ):ab,ti,kw OR (whole body vibration exercise ):ab,ti,kw OR (stretching ):ab,ti,kw OR (body mind exercise ):ab,ti,kw OR (Yoga ):ab,ti,kw OR (pilates ):ab,ti,kw OR (Tai Chi ):ab,ti,kw OR (Taijiquan ):ab,ti,kw OR (Health

Qigong ):ab,ti,kw OR (Yijinjing ):ab,ti,kw OR (Wuqinxi ):ab,ti,kw OR (Liuzijue ):ab,ti,kw OR (Baduanjin ):ab,ti,kw OR (multicomponent exercise ):ab,ti,kw OR (Equine-Assisted Therapy ):ab,ti,kw OR (Animal Assisted Therapy ):ab,ti,kw

#10：Child OR Adolescent OR Young Adult OR Minor

#11：(Children ):ab,ti,kw OR (Children ):ab,ti,kw OR (Child ):ab,ti,kw OR (Adolescent ):ab,ti,kw OR (Young Adult ):ab,ti,kw OR (Minor ):ab,ti,kw OR (Adolescen ):ab,ti,kw OR (Teen ):ab,ti,kw OR (Teenager ):ab,ti,kw OR (Youth ):ab,ti,kw OR (Minors ):ab,ti,kw OR (Minor ):ab,ti,kw OR (Child ):ab,ti,kw OR (Kid ):ab,ti,kw OR (Girl ):ab,ti,kw OR (Boy ):ab,ti,kw OR (Under age ):ab,ti,kw OR (Underage ):ab,ti,kw OR (Young people):ab,ti,kw OR (young person ):ab,ti,kw OR (Prepubescen ):ab,ti,kw OR (Pubescen ):ab,ti,kw OR (Young Adult ):ab,ti,kw OR (School age ):ab,ti,kw OR (Preschool ):ab,ti,kw OR (Student ):ab,ti,kw

#12：#1 OR #2

#13：#3 OR #4 #5 OR #6 #7 OR #8 OR #9

#14：#10 OR #11

#15：#12 AND #13 AND #14

Embase 2357

#1: attention AND deficit AND hyperactivity AND disorder

#2: 'attention deficit hyperactivity disorder':ab,ti OR 'attention-deficit/hyperactivity disorder':ab,ti OR 'ADHD':ab,ti OR 'disorder, attention deficit hyperactivity':ab,ti

#3：sports

#4：'sport':ab,ti OR 'athletics':ab,ti OR 'athletic':ab,ti

#5：exercise AND therapy

#6：'remedial exercise':ab,ti OR 'exercise, remedial':ab,ti OR 'exercises, remedial':ab,ti OR 'remedial exercises':ab,ti OR 'therapy, exercise':ab,ti OR 'exercise therapies':ab,ti OR 'therapies, exercise':ab,ti OR 'rehabilitation exercise':ab,ti OR 'exercise, rehabilitation':ab,ti OR 'exercises, rehabilitation':ab,ti OR 'rehabilitation exercises':ab,ti

#7：exercise

#8：'exercises':ab,ti OR 'physical activity':ab,ti OR 'activities, physical':ab,ti OR 'activity, physical':ab,ti OR 'physical activities':ab,ti OR 'exercise, physical':ab,ti OR 'exercises, physical':ab,ti OR 'physical exercise':ab,ti OR 'physical exercises':ab,ti OR 'acute exercise':ab,ti OR 'acute exercises':ab,ti OR 'exercise, acute':ab,ti OR 'exercises, acute':ab,ti OR 'exercise, isometric':ab,ti OR 'exercises, isometric':ab,ti OR 'isometric exercises':ab,ti OR 'isometric exercise':ab,ti OR 'exercise, aerobic':ab,ti OR 'aerobic exercise':ab,ti OR 'aerobic exercises':ab,ti OR 'exercises, aerobic':ab,ti OR 'exercise training':ab,ti OR 'exercise trainings':ab,ti OR 'training, exercise':ab,ti OR 'trainings, exercise':ab,ti

#9：'baseball':ab,ti OR 'basketball':ab,ti OR 'bicycling':ab,ti OR 'boxing':ab,ti OR 'cricket sport':ab,ti OR 'football':ab,ti OR 'golf':ab,ti OR 'gymnastics':ab,ti OR 'hockey':ab,ti OR 'martial arts':ab,ti OR 'mountaineering':ab,ti OR 'racquet sports':ab,ti OR 'return to sport':ab,ti OR 'rugby':ab,ti OR 'running':ab,ti OR 'skating':ab,ti OR 'snow sports':ab,ti OR 'soccer':ab,ti OR 'train':ab,ti OR 'fitness':ab,ti OR 'aerobic':ab,ti OR 'walking':ab,ti OR 'high intensity interval':ab,ti OR 'resistance':ab,ti OR 'core stability':ab,ti OR 'dance':ab,ti OR 'breathing exercise':ab,ti OR 'virtual reality exercise':ab,ti OR 'whole body vibration exercise':ab,ti OR 'stretching':ab,ti OR 'body mind exercise':ab,ti OR 'yoga':ab,ti OR 'pilates':ab,ti OR 'tai chi':ab,ti OR 'taijiquan':ab,ti OR 'health qigong':ab,ti OR 'yijinjing':ab,ti OR 'wuqinxi':ab,ti OR 'liuzijue':ab,ti OR 'baduanjin':ab,ti OR 'multicomponent exercise':ab,ti OR 'equine-assisted therapy':ab,ti OR 'animal assisted therapy':ab,ti

#10：'child'/exp OR child

#11：'children':ab,ti OR 'adolescent':ab,ti OR 'adolescen':ab,ti OR 'teen':ab,ti OR 'teenager':ab,ti OR 'youth':ab,ti OR 'minors':ab,ti OR 'minor':ab,ti OR 'child':ab,ti OR 'kid':ab,ti OR 'girl':ab,ti OR 'boy':ab,ti OR 'under age':ab,ti OR 'underage':ab,ti OR 'young people':ab,ti OR 'young person':ab,ti OR 'prepubescen':ab,ti OR 'pubescen':ab,ti OR 'young adult':ab,ti OR 'school age':ab,ti OR 'preschool':ab,ti OR 'student':ab,ti

#12：#1 OR #2

#13：#3 OR #4 OR #5 OR #6 OR #7 OR #8 OR #9

#14：#10 OR #11

#15：#12 AND #13 AND #14

**Figure 1 Convergence diagnosis**

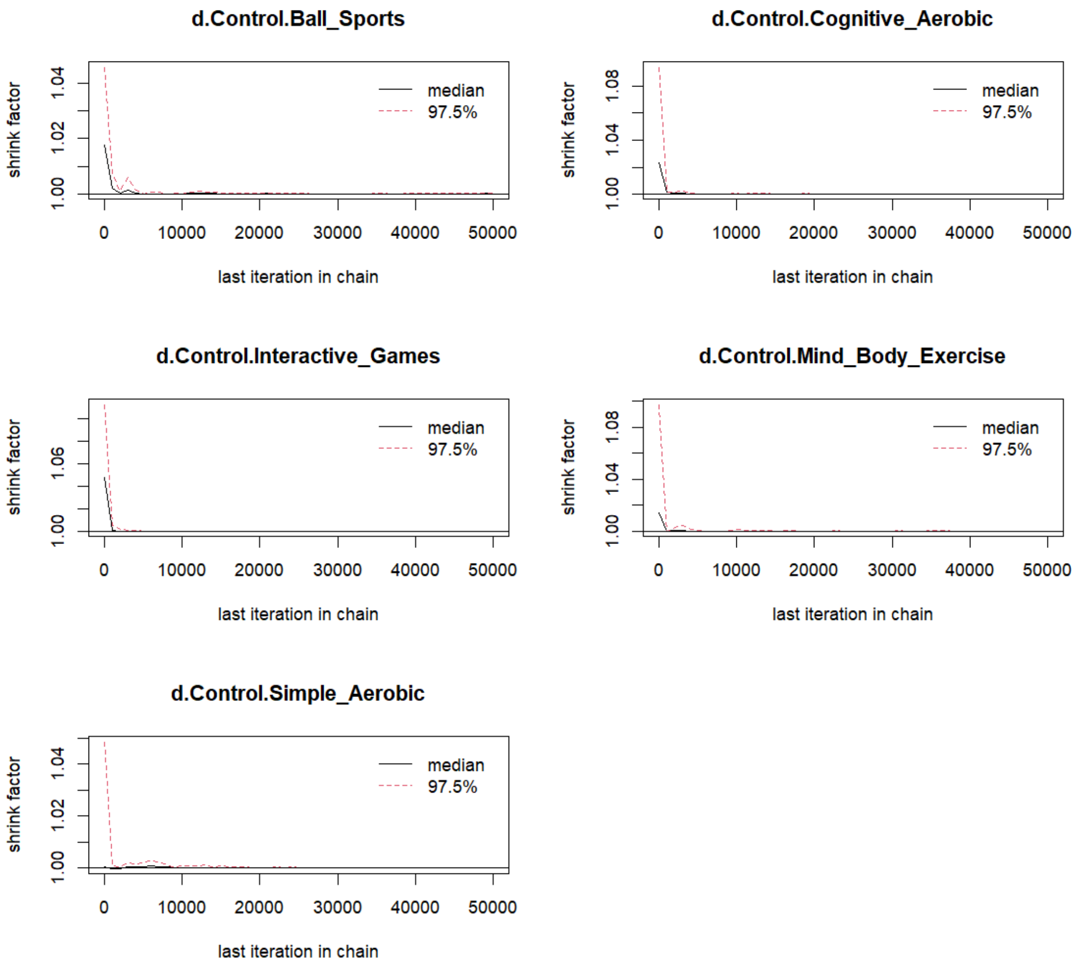

**Figure 2** Trajectory and density plots for consistency model testing of various intervention measures

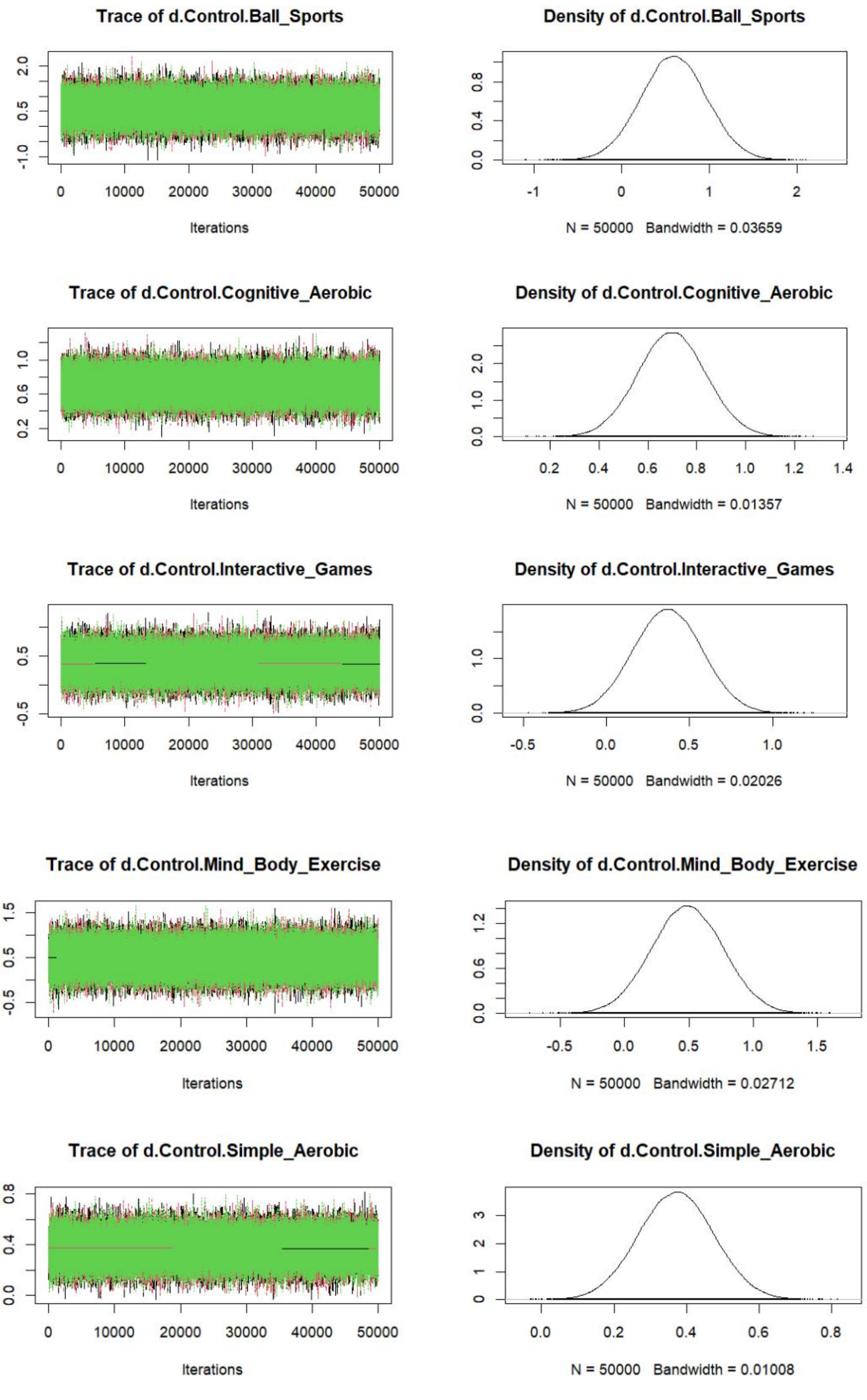

**Figure 3** Consistency model and inconsistency model

```
1. Empirical mean and standard deviation for each variable,
   plus standard error of the mean:

                               Mean     SD  Naive SE Time-series SE
d.Ball_Sports.Control       -0.5906 0.4834 0.0010809      0.0028135
d.Cognitive_Aerobic.Control -0.7526 0.2101 0.0004697      0.0010878
d.Control.Interactive_Games  0.3655 0.2994 0.0006694      0.0014033
d.Control.Mind_Body_Exercise 0.4574 0.3602 0.0008054      0.0024110
d.Control.Simple_Aerobic     0.4013 0.1465 0.0003276      0.0007954
sd.d                         0.2728 0.1422 0.0003180      0.0018765

2. Quantiles for each variable:

                                2.5%     25%     50%     75%   97.5%
d.Ball_Sports.Control       -1.54991 -0.9020 -0.5917 -0.2759  0.3590
d.Cognitive_Aerobic.Control -1.18914 -0.8814 -0.7473 -0.6175 -0.3503
d.Control.Interactive_Games -0.23095  0.1775  0.3641  0.5545  0.9616
d.Control.Mind_Body_Exercise -0.25739  0.2227  0.4602  0.6950  1.1604
d.Control.Simple_Aerobic     0.11821  0.3066  0.3985  0.4929  0.7000
sd.d                         0.02947  0.1737  0.2622  0.3573  0.5878

-- Model fit (residual deviance):

    Dbar       pD      DIC
18.26404 10.98721 29.25125

18 data points, ratio 1.015, I^2 = 7%
```

```
1. Empirical mean and standard deviation for each variable,
   plus standard error of the mean:

                               Mean     SD  Naive SE Time-series SE
d.Control.Ball_Sports        0.5962 0.3743 0.0009665      0.0009665
d.Control.Cognitive_Aerobic  0.6983 0.1388 0.0003584      0.0003595
d.Control.Interactive_Games  0.3685 0.2072 0.0005351      0.0005328
d.Control.Mind_Body_Exercise 0.4886 0.2774 0.0007163      0.0007113
d.Control.Simple_Aerobic     0.3721 0.1032 0.0002663      0.0002656

2. Quantiles for each variable:

                                 2.5%    25%    50%    75%  97.5%
d.Control.Ball_Sports        -0.13897 0.3447 0.5960 0.8481 1.3302
d.Control.Cognitive_Aerobic   0.42704 0.6048 0.6981 0.7916 0.9715
d.Control.Interactive_Games  -0.03585 0.2285 0.3685 0.5086 0.7744
d.Control.Mind_Body_Exercise -0.05328 0.3015 0.4879 0.6771 1.0333
d.Control.Simple_Aerobic      0.17085 0.3021 0.3721 0.4416 0.5745

-- Model fit (residual deviance):

     Dbar        pD       DIC
25.988278  5.005582 30.993860

18 data points, ratio 1.444, I^2 = 35%
```

**Figure 4** loop-specific heterogeneity estimates

```
Evaluation of inconsistency using loop-specific heterogeneity estimates:
```

| Loop | IF | seIF | z_value | p_value | CI_95 | Loop_Heterog_tau2 |
|---|---|---|---|---|---|---|
| A-B-D | 0.036 | 0.517 | 0.070 | 0.944 | (0.00,1.05) | 0.064 |

**Figure 5** Funnel plots of publication bias for outcome measures of included studies

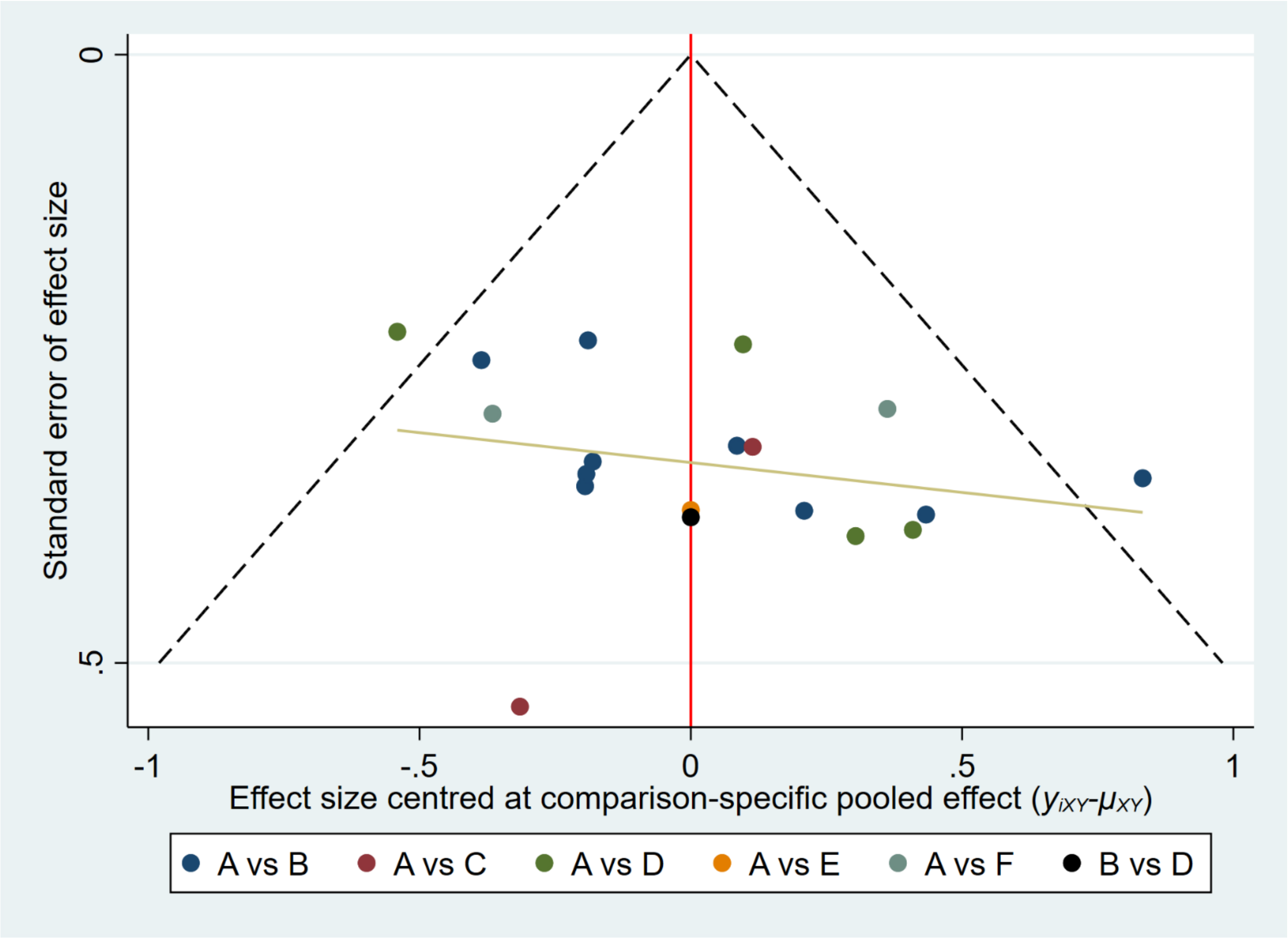

**Figure 6** Regressive analysis

```
1. Empirical mean and standard deviation for each variable,
   plus standard error of the mean:

                              Mean     SD Naive SE Time-series SE
d.Control.Ball_Sports     -4.15623 22.059 0.049325        4.47009
d.Control.Cognitive_Aerobic 13.05821 3.656 0.008176      0.14471
d.Control.Interactive_Games 7.79921 4.134 0.009245       0.13359
d.Control.Mind_Body_Exercise 0.78857 2.631 0.005883      0.04662
d.Control.Simple_Aerobic   0.75378  1.331 0.002976        0.05014
sd.d                       1.35044  2.132 0.004766        0.15691
beta[1]                   19.94860 85.702 0.191636       17.35924
beta[2]                  -21.92652  8.456 0.018908        0.34397
beta[4]                   16.70347 10.391 0.023235        0.37034
beta[5]                    4.85920 16.484 0.036860        0.40429
beta[6]                    0.02607  3.115 0.006965        0.44295

2. Quantiles for each variable:

                              2.5%      25%      50%      75%   97.5%
d.Control.Ball_Sports     -64.4288 -12.1289  -0.3990   8.2094  32.750
d.Control.Cognitive_Aerobic 6.0051 10.6722  13.0480  15.4206  20.209
d.Control.Interactive_Games -0.4009  5.4781  7.8655  10.2296  15.368
d.Control.Mind_Body_Exercise -3.9238 -0.5848  0.7933  2.1644   5.529
d.Control.Simple_Aerobic   -0.3870   0.1461   0.4181   0.8692   4.428
sd.d                        0.0721   0.3536   0.6474   1.3105   7.831
beta[1]                  -122.9484 -27.4875   5.0822  49.7910 255.141
beta[2]                   -38.6381 -27.2583 -21.8755 -16.4621  -5.492
beta[4]                    -5.1325  10.9526  16.9658  22.8826  36.003
beta[5]                   -26.6343  -4.8021   4.7900  14.3952  37.114
beta[6]                    -4.1184  -0.9558  -0.2349   0.4039   6.959

-- Model fit (residual deviance):

    Dbar       pD      DIC
48.55892 30.11433 78.67326

35 data points, ratio 1.387, I^2 = 30%

-- Regression settings:

Regression on "Allh", unrelated coefficients, "Control" as control
Input standardized: x' = (Allh - 18.02) / 39.04291
Estimates at the centering value: Allh = 18.02
```

```
1. Empirical mean and standard deviation for each variable,
   plus standard error of the mean:

                             Mean     SD Naive SE Time-series SE
d.Control.Ball_Sports     -8.9269 33.850 0.075692        8.38119
d.Control.Cognitive_Aerobic 6.8822 4.244 0.009489        0.11549
d.Control.Interactive_Games 8.0031 8.589 0.019207        0.50745
d.Control.Mind_Body_Exercise 0.4407 4.824 0.010786       0.15774
d.Control.Simple_Aerobic   1.7240  2.153 0.004815        0.06315
sd.d                       3.3203  3.150 0.007044        0.12926
beta[1]                   15.0429 51.274 0.114652       13.26426
beta[2]                   -3.1022 12.291 0.027484        0.50289
beta[4]                   10.4346 15.164 0.033907        0.98349
beta[5]                    2.9658 14.106 0.031543        0.68863
beta[6]                    1.1762  3.341 0.007470        0.27894

2. Quantiles for each variable:

                               2.5%      25%     50%    75%   97.5%
d.Control.Ball_Sports     -99.25829 -18.4025 -1.6686 10.360  46.208
d.Control.Cognitive_Aerobic -0.45369  4.1996  6.4908  9.083  16.721
d.Control.Interactive_Games -13.31667  4.2978 8.9042 12.924  23.072
d.Control.Mind_Body_Exercise -10.22638 -1.7995 0.4931 2.747  10.687
d.Control.Simple_Aerobic   -0.74890   0.2485  0.9952  2.757   7.203
sd.d                        0.09569   0.5814  2.3754  5.422  10.632
beta[1]                   -69.11305 -13.8910  3.9133 28.208 152.000
beta[2]                   -25.61495 -10.4803 -4.2182  2.778  26.590
beta[4]                   -28.36983   4.4508 12.3878 19.044  36.748
beta[5]                   -26.77552  -3.5715  2.6863  9.179  35.459
beta[6]                    -3.78482  -0.8155  0.3457  2.628  10.027

-- Model fit (residual deviance):

    Dbar       pD      DIC
49.33725 33.56113 82.89838

35 data points, ratio 1.41, I^2 = 31%

-- Regression settings:

Regression on "Min", unrelated coefficients, "Control" as control
Input standardized: x' = (Min - 43.23529) / 40.91778
Estimates at the centering value: Min = 43.23529
```

```
1. Empirical mean and standard deviation for each variable,
   plus standard error of the mean:

                              Mean      SD Naive SE Time-series SE
d.Control.Ball_Sports       0.1298  14.799  0.03309        0.74657
d.Control.Cognitive_Aerobic 11.9658  7.791  0.01742        0.12044
d.Control.Interactive_Games 3.1934   9.142  0.02044        0.03403
d.Control.Mind_Body_Exercise 1.3062  9.791  0.02189        0.14254
d.Control.Simple_Aerobic    0.2968   4.899  0.01095        0.07454
sd.d                        7.8331  10.067  0.02251        0.22024
beta[1]                   -10.5288 101.245  0.22639        9.72693
beta[2]                   -17.0344  15.391  0.03442        0.80312
beta[4]                     5.5437  14.136  0.03161        0.89390
beta[5]                    -1.0616  27.054  0.06049        1.43457
beta[6]                   -14.4210  26.815  0.05996        4.77773

2. Quantiles for each variable:

                              2.5%       25%      50%      75%   97.5%
d.Control.Ball_Sports     -34.2024  -3.83125   0.7839  4.89864  31.814
d.Control.Cognitive_Aerobic 0.2694  7.71211  10.7922 14.50961  33.033
d.Control.Interactive_Games -18.9306 1.43729  3.2595  5.02644  24.901
d.Control.Mind_Body_Exercise -22.2793 -0.13581 1.4015 2.90809  24.524
d.Control.Simple_Aerobic  -14.0262   0.03167   0.5406  1.90093   8.625
sd.d                        0.1218   0.68989   2.9401 10.24527  31.205
beta[1]                  -323.9108 -33.82670  -2.3297 25.30658 202.301
beta[2]                   -49.7067 -25.06695 -16.5947 -8.27456  12.790
beta[4]                   -30.1364   2.26660   6.2709  9.87818  36.816
beta[5]                   -53.4718  -8.34351  -1.8988  4.88791  53.383
beta[6]                   -97.1033 -10.26310  -1.1789 -0.02043   3.452

-- Model fit (residual deviance):

    Dbar       pD      DIC
45.86505 36.12628 81.99133

35 data points, ratio 1.31, I^2 = 26%

-- Regression settings:

Regression on "Times", unrelated coefficients, "Control" as control
Input standardized: x' = (Times - 2.176471) / 2.370158
Estimates at the centering value: Times = 2.176471
```

```
1. Empirical mean and standard deviation for each variable,
   plus standard error of the mean:

                              Mean     SD Naive SE Time-series SE
d.Control.Ball_Sports       2.7753 23.086  0.05162        2.88667
d.Control.Cognitive_Aerobic 13.6916 11.009 0.02462       0.38618
d.Control.Interactive_Games 4.7528 11.168  0.02497        0.15510
d.Control.Mind_Body_Exercise -0.0244 11.584 0.02590       0.21441
d.Control.Simple_Aerobic    5.1034  8.523  0.01906        0.07811
sd.d                        8.8748 11.678  0.02611        0.09588
beta[1]                    -4.2876 40.075  0.08961        7.10680
beta[2]                   -11.6081 16.616  0.03715        1.51137
beta[4]                     7.3665 14.879  0.03327        1.12518
beta[5]                     4.3444 19.468  0.04353        1.77446
beta[6]                    21.7606 39.057  0.08733        4.77727

2. Quantiles for each variable:

                              2.5%      25%      50%    75%  97.5%
d.Control.Ball_Sports     -44.9374  -9.0882   1.8145 14.234  56.01
d.Control.Cognitive_Aerobic -0.5608  8.0586  11.1439 15.633  46.18
d.Control.Interactive_Games -23.2725  2.5856  4.9074  7.218  31.68
d.Control.Mind_Body_Exercise -30.5357 -2.3599 0.5247  3.346  25.39
d.Control.Simple_Aerobic   -0.6836   0.3206   1.0941  5.099  29.85
sd.d                        0.1205   0.7217   2.5685 15.575  31.61
beta[1]                   -94.5712 -25.2506  -1.9862 18.228  72.66
beta[2]                   -52.5051 -17.9368 -12.0642 -5.682  25.87
beta[4]                   -30.4313   3.6401   8.1076 12.525  37.74
beta[5]                   -33.5442  -3.9492   2.7554  9.789  58.95
beta[6]                    -3.1576  -0.5185   0.2779 18.786 116.20

-- Model fit (residual deviance):

    Dbar       pD      DIC
45.86443 35.78641 81.65085

35 data points, ratio 1.31, I^2 = 26%

-- Regression settings:

Regression on "Week", unrelated coefficients, "Control" as control
Input standardized: x' = (Week - 7.294118) / 10.53007
Estimates at the centering value: Week = 7.294118
```

```
1. Empirical mean and standard deviation for each variable,
   plus standard error of the mean:

                              Mean      SD Naive SE Time-series SE
d.Control.Ball_Sports      -1.9403  48.924  0.10940        8.86064
d.Control.Cognitive_Aerobic 9.6030   8.833  0.01975        0.05071
d.Control.Interactive_Games 2.8178  10.671  0.02386        0.02918
d.Control.Mind_Body_Exercise 0.7352 12.809  0.02864        0.43954
d.Control.Simple_Aerobic    1.1349   5.075  0.01135        0.02834
sd.d                        9.3911  11.631  0.02601        0.06964
beta[1]                     3.3705  55.265  0.12358       12.84015
beta[2]                     6.6419  11.091  0.02480        0.34967
beta[4]                   -42.7174 124.756  0.27896        7.73044
beta[5]                    -1.3549  25.615  0.05728        3.47030
beta[6]                   -20.9291  40.767  0.09116        2.06306

2. Quantiles for each variable:

                              2.5%      25%      50%    75%   97.5%
d.Control.Ball_Sports    -108.6410 -24.3553   1.2709 19.542 114.575
d.Control.Cognitive_Aerobic -1.9198  5.2145   7.1812 10.881  35.527
d.Control.Interactive_Games -23.4494 0.7118   2.7182  4.810  29.415
d.Control.Mind_Body_Exercise -29.1802 -2.9562 0.4250  4.022  32.908
d.Control.Simple_Aerobic  -12.2088   0.2183   0.9273  2.560  12.579
sd.d                        0.1425   1.2531   3.5341 16.204  31.581
beta[1]                  -130.9335 -20.4424  -0.4145 25.784 129.808
beta[2]                   -22.0660   3.1007   7.7304 11.924  26.201
beta[4]                  -239.6006 -69.9494 -23.4403  5.587  97.902
beta[5]                   -51.6307 -11.1045  -2.6570  6.289  70.707
beta[6]                  -112.7453 -17.0882   0.2952  2.821   9.127

-- Model fit (residual deviance):

    Dbar       pD      DIC
44.56196 35.67654 80.23849

35 data points, ratio 1.273, I^2 = 24%

-- Regression settings:

Regression on "Sex", unrelated coefficients, "Control" as control
Input standardized: x' = (Sex - 0.8235294) / 0.2092775
Estimates at the centering value: Sex = 0.8235294
```

```
1. Empirical mean and standard deviation for each variable,
   plus standard error of the mean:

                              Mean     SD Naive SE Time-series SE
d.Control.Ball_Sports       2.7450  28.08  0.06279        1.64084
d.Control.Cognitive_Aerobic 11.5416 17.04  0.03809        1.09090
d.Control.Interactive_Games -0.5746 21.80  0.04876        2.32279
d.Control.Mind_Body_Exercise 1.1222 14.09  0.03150        0.03483
d.Control.Simple_Aerobic   -6.4313  13.70  0.03062        0.03653
sd.d                       16.5557  10.99  0.02457        0.18727
beta[1]                     5.8316  62.93  0.14072       10.62653
beta[2]                    10.0448  29.34  0.06560        3.32034
beta[4]                    55.0959 281.92  0.63039       41.26631
beta[5]                    -0.5901  34.53  0.07721        1.81869
beta[6]                    52.1253  67.34  0.15058        2.22479

2. Quantiles for each variable:

                             2.5%       25%      50%    75%    97.5%
d.Control.Ball_Sports     -56.503 -11.25763  1.30322 16.227   66.101
d.Control.Cognitive_Aerobic -30.065 3.60297 13.41778 22.003   40.942
d.Control.Interactive_Games -63.554 -6.32134 1.94271 9.167    36.828
d.Control.Mind_Body_Exercise -30.464 -4.38823 1.15256 6.655   32.540
d.Control.Simple_Aerobic  -40.253 -12.46959 -0.26483  2.743    8.257
sd.d                        1.616   5.68735 14.91412 30.326   31.868
beta[1]                  -144.078 -19.92626  3.74354 36.242  129.248
beta[2]                   -64.308  -3.41498 11.90689 27.003   66.379
beta[4]                  -227.454 -27.45328  3.03988 39.553 1055.169
beta[5]                   -76.171 -16.38405 -0.08317 15.159   72.034
beta[6]                    -5.743  -0.06015 20.50039 75.550  173.321

-- Model fit (residual deviance):

    Dbar       pD      DIC
37.31572 35.17542 72.49115

35 data points, ratio 1.066, I^2 = 9%

-- Regression settings:

Regression on "Age", unrelated coefficients, "Control" as control
Input standardized: x' = (Age - 10.24235) / 3.691981
Estimates at the centering value: Age = 10.24235
```

**Figure 7** RoB2 literature publication bias

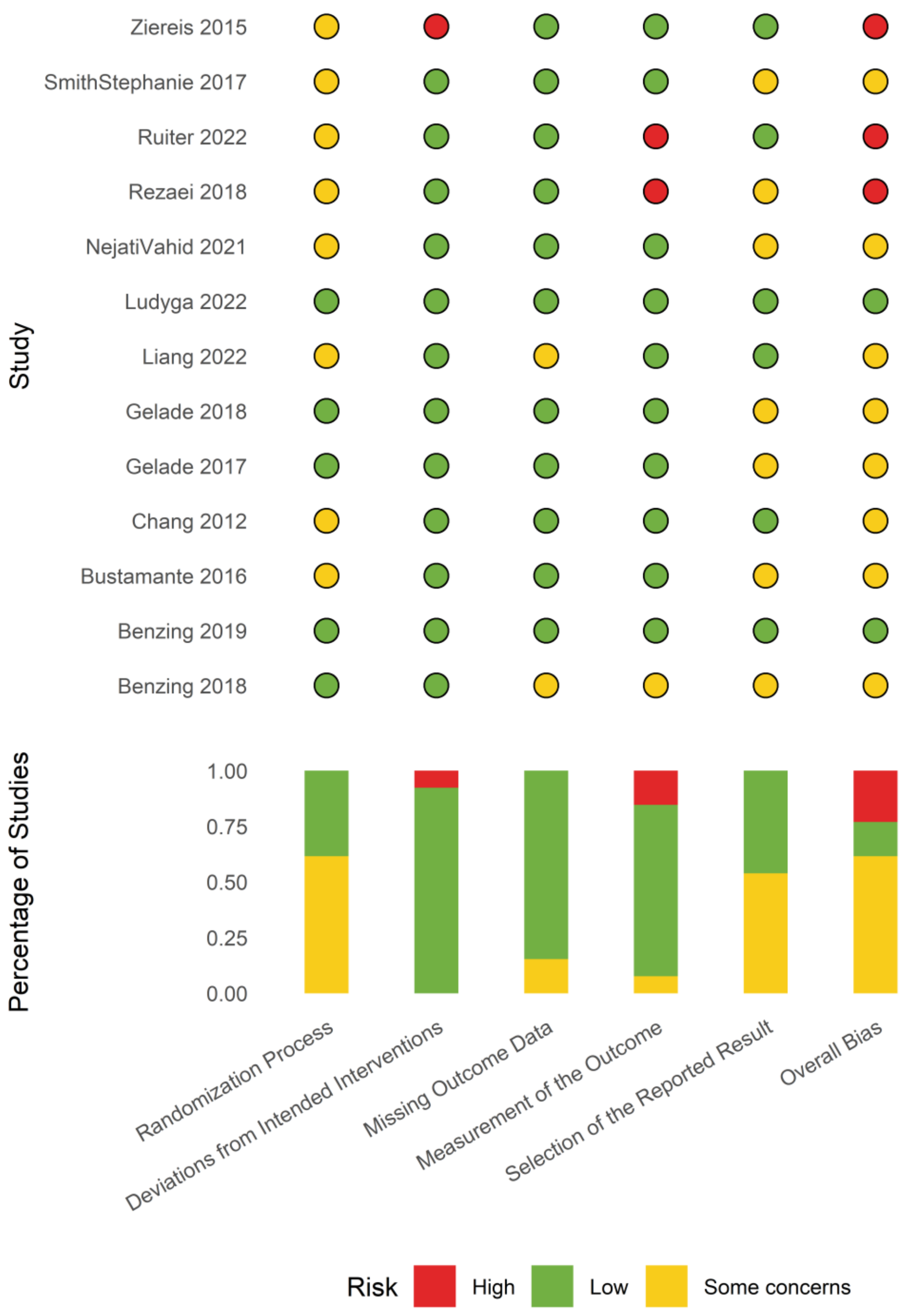

**Figure 8** ROBINS-I V2 literature publication bias

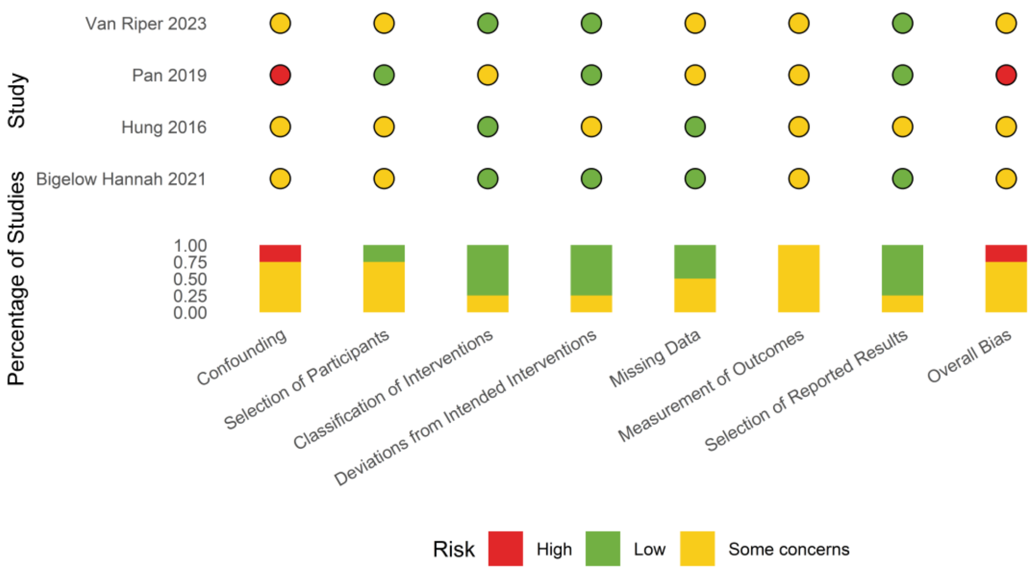

Table 1 MINORS literature quality evaluation table

| study | A1 | A2 | A3 | A4 | A5 | A6 | A7 | A8 | A9 | A10 | A11 | A12 | Total score |
|---|---|---|---|---|---|---|---|---|---|---|---|---|---|
| Van Riper 2023 | 2 | 0 | 2 | 2 | 0 | 0 | 0 | 0 | 2 | 2 | 2 | 2 | 14 |
| Bigelow Hannah 2021 | 2 | 0 | 2 | 2 | 0 | 1 | 0 | 0 | 1 | 2 | 2 | 2 | 14 |
| | | | | | | | | | | | | | |
| Pan 2019 | 2 | 0 | 2 | 2 | 0 | 2 | 0 | 0 | 1 | 2 | 2 | 2 | 15 |
| Hung 2016 | 2 | 0 | 2 | 2 | 0 | 2 | 0 | 0 | 2 | 2 | 2 | 2 | 16 |

MINORS, Methodological Index for Non-Randomized Studies

A1: A clearly stated aim, A2: Inclusion of consecutive patients, A3: Prospective collection of data, A4: Endpoints appropriate to the aim of the study, A5: Unbiased assessment of the study endpoint, A6: Follow-up period appropriate to the aim of the study, A7: Loss to follow-up less than 5%, A8: Prospective calculation of the study size, A9: An adequate control group, A10: Contemporary groups, A11: Baseline equivalence of groups, and A12: Adequate statistical analyses.

Table 2 PEDro literature Quality evaluation

| Study | A1 | A2 | A3 | A4 | A5 | A6 | A7 | A8 | A9 | A10 | A11 | Total score |
|---|---|---|---|---|---|---|---|---|---|---|---|---|
| Ludyga 2022 | 1 | 1 | 0 | 1 | 0 | 0 | 0 | 1 | 0 | 1 | 1 | 5 |
| Liang 2022 | 1 | 1 | 0 | 1 | 0 | 0 | 0 | 0 | 0 | 1 | 1 | 4 |
| Nejati 2021 | 1 | 1 | 0 | 1 | 0 | 0 | 0 | 1 | 1 | 1 | 1 | 6 |
| Smith 2020 | 1 | 1 | 0 | 1 | 0 | 0 | 0 | 1 | 1 | 1 | 1 | 6 |
| Gelade 2018 | 1 | 1 | 1 | 1 | 0 | 0 | 0 | 1 | 1 | 1 | 1 | 7 |
| Benzing 2019 | 1 | 1 | 1 | 1 | 0 | 0 | 0 | 1 | 1 | 1 | 1 | 7 |
| Rezaei 2018 | 1 | 1 | 0 | 1 | 0 | 0 | 0 | 1 | 1 | 1 | 1 | 6 |
| Benzing 2018 | 1 | 1 | 1 | 1 | 0 | 0 | 0 | 1 | 1 | 1 | 1 | 7 |
| Gelade 2017 | 1 | 1 | 0 | 1 | 1 | 0 | 1 | 1 | 1 | 1 | 1 | 8 |
| Bustamante 2016 | 1 | 1 | 0 | 1 | 0 | 0 | 1 | 1 | 1 | 1 | 1 | 7 |
| Ruiter 2022 | 1 | 1 | 0 | 1 | 0 | 0 | 0 | 1 | 1 | 1 | 1 | 6 |
| Chang 2012 | 1 | 1 | 0 | 1 | 0 | 0 | 0 | 1 | 1 | 1 | 1 | 6 |
| Ziereis 2015 | 1 | 1 | 1 | 1 | 0 | 0 | 0 | 1 | 1 | 1 | 1 | 7 |

PEDro, Physiotherapy Evidence Database

Al: participant selection criteria specified; A2: random assignment of participants to groups; A3: hidden assignment; A4: groups were similar at baseline; A5: all participants were blind; A6: all therapists were blind; A7: all assessors were blind; A8: measurement of at least one of the main outcomes was obtained from more than 85%of the participants.A9: intention-to-treat analysis was conducted; A10: results of statistical comparisons between groups for at least one main outcome were reported; Al1: the study reported point and variability measures for at least one main outcome
